# SLC6A14 Drives Mitochondrial Fusion and Oxidative Phosphorylation to Promote Cancer Stemness and Early‐Onset of Breast Cancer

**DOI:** 10.1002/advs.202510811

**Published:** 2025-09-17

**Authors:** Dai‐Wei Hu, Chih‐Hao Huang, Yu‐Hao He, Ya‐Ling Wei, Shu‐Wei Hu, Fang‐Ju Cheng, Thanh Kieu Huynh, Bo‐Rong Chen, Bo‐Wei Wang, Li‐Chi Kuan, Der‐Yen Lee, Ming‐Hsin Yeh, Ya‐Jen Chang, Liang‐Chih Liu, Mien‐Chie Hung, Wei‐Chien Huang

**Affiliations:** ^1^ Graduate Institute of Biomedical Science China Medical University Taichung 404 Taiwan; ^2^ Center for Molecular Medicine China Medical University Hospital Taichung 406 Taiwan; ^3^ School of Medicine College of Medicine China Medical University Taichung 404 Taiwan; ^4^ Division of Breast Surgery China Medical University Hospital Taichung 404 Taiwan; ^5^ Department of Biological Science and Technology China Medical University Taichung 404 Taiwan; ^6^ Graduate Institute of Integrated Medicine China Medical University Taichung 404 Taiwan; ^7^ Department of Surgery Chung Shan Medical University Hospital Taichung 402 Taiwan; ^8^ Institute of Medicine School of Medicine Chung Shan Medical University Taichung 402 Taiwan; ^9^ Institute of Biomedical Sciences Academia Sinica Taipei 115 Taiwan; ^10^ Department of Medical Laboratory Science and Biotechnology Asia University Taichung 413 Taiwan; ^11^ Cancer Biology and Precision Therapeutic Center China Medical University Taichung 406 Taiwan; ^12^ Department of Medical Research China Medical University Hsinchu Hospital Hsinchu 302 Taiwan

**Keywords:** cancer stemness, early‐onset breast cancer, mitochondria dynamics, plasticizer, SLC6A14

## Abstract

Early‐onset breast cancer (EOBC), diagnosed before the age of 45, is associated with poor therapeutic outcomes and limited survival, yet the underlying mechanisms remain poorly defined. Identifying environmental risk factors and actionable therapeutic targets is an urgent clinical need. Notably, the largest survival gap between younger and older patients occurs in luminal breast cancer, implicating potential endocrine disruption. Here, an association is identified between elevated levels of di(2‐ethylhexyl)phthalate (DEHP) in hair, a widely used endocrine‐disrupting plasticizer, and earlier age at diagnosis of breast cancer. Mechanistically, DEHP exposure promotes tumor initiation by enhancing cancer stemness through mitochondrial fusion and glutamine‐driven oxidative phosphorylation. DEHP upregulates the glutamine transporter SLC6A14 to enhance glutamine uptake, while suppressing mitochondrial fission factor (MFF), which exacerbates mitochondrial fusion. High SLC6A14 expression correlates with cancer stemness signatures and earlier onset in patient cohorts. Inhibition of SLC6A14 reduces stemness, impairs tumor growth, and sensitizes tumors to chemotherapy. Collectively, the findings uncover a novel environmental‐metabolic axis linking plasticizer exposure to EOBC and establish SLC6A14 as a promising metabolic vulnerability. These results provide a strong preclinical rationale for targeting SLC6A14 in young breast cancer patients and offer new insights into mitigating the oncogenic impact of environmental pollutants.

## Graphical abstract

Elevated levels of the plasticizer DEHP associate with earlier breast cancer diagnosis. DEHP enhances glutamine‐powered energy production via the transporter SLC6A14 and pushes mitochondria toward fusion, driving tumor initiation by boosting cancer stemness. Blocking SLC6A14 lowers stemness, slows tumors, and improves chemotherapy response. The study reveals a plasticizer–metabolism axis and nominates SLC6A14 as a therapeutic target in young patients.

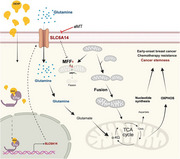

## 1 Introduction

The global incidence of early‐onset breast cancers (EOBCs), usually diagnosed in patients younger than 45 years old, has increased by 16% since the 1990s^[^ ^1^ ^]^ and raised unique challenges, including difficult medical, fertility preservation, pregnancy considerations, psychosocial, financial, and health issues.^[^ ^2^, ^3^, ^4^, ^5^ ^]^ These young patients with EOBC showed a higher occurrence of distant metastasis and mortality than their older counterparts.^[^ ^6^ ^]^ Despite advancements in early detection and treatment for breast cancer, these young patients experience poorer prognosis and less effective therapeutic responses compared to older patients.^[^ ^7^ ^]^ These observations for EOBC are of grave concern and indicate the importance of assessing this disease's unique risk and mechanism. Identification of the potential therapeutic target is an urgent demand for this disease.

Although genetic predisposition was the focus of many EOBC‐associated studies, less than 12% of women under 40 diagnosed with breast cancer carry the BRCA1 or BRCA2 mutations.^[^ ^8^ ^]^ The overwhelming majority of EOBC, similar to those in their older counterparts, were diagnosed in those without a known germline genetic mutation or family history of breast cancer.^[^ ^7^ ^]^ Most EOBC cases are hormone receptor‐positive subtypes,^[^ ^9^ ^]^ including luminal A (50.3%) and luminal B (27.6%).^[^ ^10^ ^]^ Notably, younger breast cancer patients with luminal B subtype exhibited less satisfactory outcomes^[^ ^11^ ^]^ and a higher recurrence rate.^[^ ^12^ ^]^ Surprisingly, the most significant breast cancer survival gap between younger and older patients occurs with the less‐aggressive luminal breast cancers but not aggressive tumor types, such as triple‐negative and human epidermal growth factor receptor 2 (HER2)‐enriched tumors,^[^ ^8^ ^]^ implying the involvement of endocrine disruption in the tumorigenesis and malignancy of EOBC. There is a clear need to understand why breast cancers behave differently and have much less favorable outcomes in younger women.

Besides genetic predisposition, several lifestyle and reproductive risk factors have been implicated in developing breast cancer in young women.^[^ ^13^ ^]^ Plasticizers are chemical additives used to aid manufacturing and increase the flexibility of plastic products in diverse fields, including medicine, cosmetics, electronics, food packaging, and construction. Di(2‐ethylhexyl)phthalate (DEHP), acting as an endocrine disruptor and structurally resembling human estrogen,^[^ ^14^ ^]^ is the predominant phthalate plasticizer extensively used in manufacturing plastic products, and shows detectable levels in water, various fish species in rivers,^[^ ^15^ ^]^ and even in the inhaled particulate matter^[^ ^16^ ^]^ in the plastic life cycle. Studies have reported that phthalate metabolites were detected in over 70% of the general U.S population^[^ ^17^ ^]^ and 99% of the general Korean adult population^[^ ^18^ ^]^ in urine samples. DEHP has a high absorption potential, particularly in younger individuals,^[^ ^19^ ^]^ and has been detected in fetal placenta^[^ ^20^ ^]^ and hair.^[^ ^21^ ^]^ Moreover, DEHP is also known to cross the placenta^[^ ^20^ ^]^ and is detectable in human milk.^[^ ^22^ ^]^ These observations indicate a near‐universal plasticizer exposure and its harmful impact on threatening the health of more than 2 billion people,^[^ ^23^ ^]^ especially in young children.^[^ ^24^ ^]^

Although DEHP is excreted in urine within 24–48 h,^[^ ^25^ ^]^ chronic exposure to DEHP has been shown to cause metabolic diseases, including non‐alcoholic fatty liver disease (NAFLD) and obesity.^[^ ^26^ ^]^ In a cohort of breast cancer patients, higher urinary Σ4MEHP (sum of DEHP metabolites) was associated with tumor progression, increased recurrence risk, and clinicopathologic features including estrogen receptor (ER) and HER2 status.^[^ ^27^ ^]^ Schoolchildren have a higher daily phthalate intake in East Asia than in Western countries,^[^ ^28^ ^]^ raising a potential explanation for the higher occurrence of EOBC in East Asia.^[^ ^29^ ^]^ Therefore, it is imperative to investigate whether and how plasticizers accelerate the early‐onset and drug resistance of breast cancer to develop effective therapies for EOBC.

Tumor bioenergetics are not a strict Warburg on/off model: glycolysis and mitochondrial oxidative phosphorylation (OXPHOS) typically coexist, with their balance tuned by oncogenic programs, nutrient and oxygen supply, and treatment stress. Mitochondria remain crucial for ATP generation, redox control, and precursor supply via glutamine anaplerosis and, when respiration is constrained, reductive carboxylation to acetyl‐CoA/lipids.^[^ ^30^ ^]^ Cancer stem cells (CSCs), responsible for self‐renewal, differentiation, and tumor initiation and regrowth,^[^ ^31^, ^32^ ^]^ are likewise metabolically heterogeneous: many therapy‐tolerant states exhibit elevated mitochondrial mass/OXPHOS, fatty‐acid oxidation, and fused mitochondrial networks, whereas other states favor glycolysis, with program shifts with niche signals and cell‐state changes.^[^ ^33^ ^]^ The populations of CSCs are elevated in the tumors of younger breast cancer patients,^[^ ^34^, ^35^ ^]^ suggesting that increased cancer stemness may contribute to the early onset and poor prognosis of breast cancer.

“Mitostemness” traits have been designated the mitochondria‐dependent signaling functions, including mitochondrial fission/fusion dynamics, that regulate the maintenance of CSC characteristics.^[^ ^36^ ^]^ To control the mitochondrial quality by eliminating dysfunctional mitochondria through mitophagy, mitochondrial fission splits a single mitochondrion into two short or fragmented daughter mitochondria. This process is primarily initiated by the recruitment of dynamin‐related protein 1 (DRP1) to its adapter receptors, including mitochondrial fission factor (MFF), on the outer mitochondrial membrane (OMM), followed by the oligomerization and GTPase activation of DRP1 to form a helical structure.^[^ ^37^ ^]^ To maintain or enhance mitochondrial function, mitochondrial fusion exchanges the intramitochondrial contents between two closely contacted mitochondria, forming an elongated and interconnected mitochondrial network. The fusion process is mainly mediated by mitofusins 1 and 2 (MFN1, MFN2) on OMM and optic atrophy 1 (OPA1) on the inner mitochondrial membrane (IMM).^[^ ^38^ ^]^ Depending on the tissue type of tumors, CSC stemness and self‐renewal can be enhanced by both mitochondrial fusion and fission.^[^ ^39^ ^]^ However, it remains unclear whether the imbalance of mitochondrial dynamics is involved in the early onset of breast cancer.

In the current study, exposure to DEHP has been found to be associated with the early onset of breast cancer in patients and was demonstrated to promote the early tumorigenesis of breast cancer in mouse models. Mechanistically, DEHP upregulates glutamine transporter SLC6A14 to enhance glutamine uptake and to promote mitochondrial fusion via MFF suppression for fueling the metabolic requirement of CSC maintenance. Inhibition of SLC6A14 repressed DEHP‐associated mitochondrial fusion and oxidative phosphorylation, CSC activity, and tumor progression. Therefore, the upregulation of mitochondria fusion and oxidative phosphorylation contributes to plasticizer‐associated early onset of breast cancer in a SLC6A14‐dependent manner, and targeting SLC6A14 is revealed as a potential therapeutic strategy for EOBC patients.

## 2 Result

### 2.1 DEHP Facilitates the Early Onset of Breast Cancer

**Figure 1:**
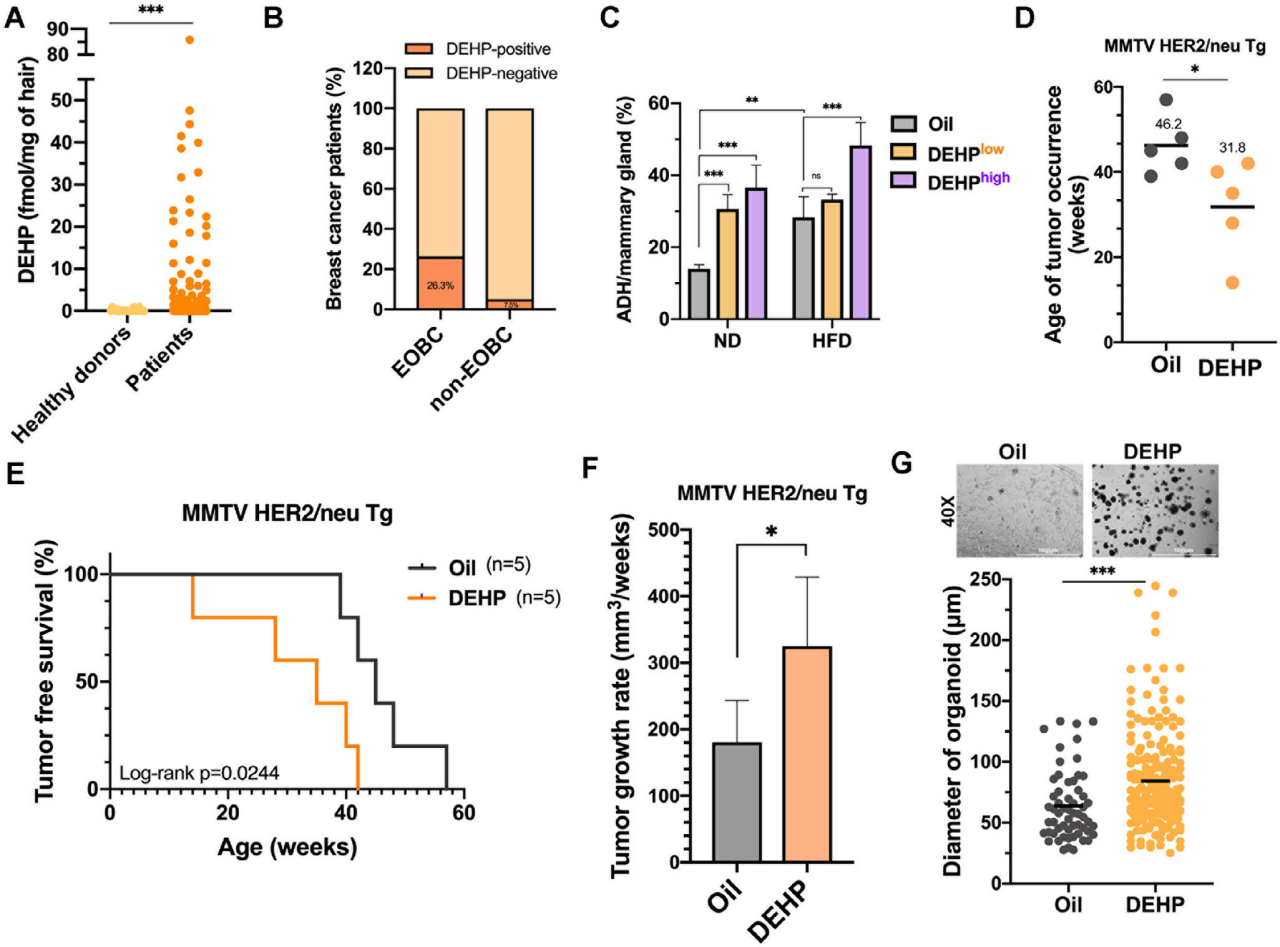
Plasticizer DEHP promotes the early onset of breast cancer in vivo. A) The hair level of DEHP from 20 healthy donors and patients with breast cancer was analyzed in LC‐MS/MS analysis. B) The percentage of DEHP+ cases in EOBC and non‐EOBC patients. C) The percentage of ADH lesions per mammary gland was determined with H&E and CK5/6 staining (n>3 mice in each group). D–G) HER2‐Tg mice were exposed to 0.21 mg kg^−1^ of DEHP for 1 year. The average time to the tumor occurrence was shown in (D). The tumor‐free survival rate was shown in (E). The tumor growth rate was calculated as the change in tumor sizes per week. A p‐value was calculated by using the log‐rank (Mantel‐Cox) test in (F). The diameter of organoids established from the tumors of HER2‐Tg mice is shown in (G). *n = 5* per group. Data were shown as the mean ± SD. ns. not significant; ^∗^ *p* < 0.05; ^∗∗^ *p* < 0.01; ^∗∗∗^ *p* < 0.001 versus the control group, Student's *t*‐test (A, D, F, and G) and two‐way ANOVA followed by Tukey's multiple comparisons test (C).

Exposure to plasticizers, especially DEHP, has been considered a risk factor for overall breast cancer,^[^ ^40^ ^]^ but its role in initiating the early onset of breast cancer remains unclear. EOBC patients, diagnosed at 45 or younger, account for 36.2% of 105 enrolled patients (Figure S1A, Supporting Information) and showed comparable molecular subtypes (Figure S1B, Supporting Information). To assess the association between long‐term DEHP exposure and the early initiation of breast cancer, we measured the DEHP level accumulated in the hair of breast cancer patients by using mass spectrum analysis.^[^ ^21^ ^]^ The average hair levels of DEHP were much higher in breast cancer patients (1.46 fmol mg^−1^) than in healthy donors (0.23fmol mg^−1^) (**Figure** **1**A). DEHP‐positive cases, whose hair DEHP level was over 10‐fold of the average in healthy donors (>2.3 fmol mg^−1^), were found in 14.3% of overall patients (Figure S1C, Supporting Information). A higher percentage of EOBC cases was observed in the DEHP‐positive group (66.7%) compared to the DEHP‐negative group (31.1%) (Figure S1D, Supporting Information). The percentage of DEHP‐positive cases (Figure 1B) and average hair DEHP level (Figure S1E, Supporting Information) were significantly higher in EOBC than in non‐EOBC. These findings suggested that exposure to DEHP is a critical risk factor for early‐onset breast cancer.

Given that obesity is another lifestyle risk factor for breast cancer,^[^ ^41^ ^]^ we next investigated the effect of DEHP exposure with a high‐fat diet (HFD) on breast cancer incidence in the Crl:CD‐1 (ICR) mice, which are prone to obesity‐related breast cancer.^[^ ^42^ ^]^ To mimic human exposure levels, the DEHP doses were selected according to reported concentrations in contaminated food (1.2 ppm)^[^ ^43^ ^]^ and the highest tolerable daily intake (8.4 mg/60 kg).^[^ ^44^ ^]^ By using human‐equivalent dose (HED) calculations based on body surface area,^[^ ^45^ ^]^ mice were therefore treated with 0.21 mg kg^−1^ (low dose) and 1.73 mg kg^−1^ (high dose) DEHP. Although no palpable tumor was detected after treatments for 1 year, DEHP‐exposed mice, subjected to either normal diets (ND) or HFD, elicited atypical hyperplasia of epithelial cells lining mammary ducts, similar to human precancerous lesion atypical ductal hyperplasia (ADH) (Figure 1C and Figure S1F, Supporting Information). This data suggests that DEHP exposure alone is sufficient to initiate ADH formation.

We further investigated the role of DEHP exposure in facilitating tumor initiation and progression in an oncogene‐driven tumor model. Although the MMTV‐HER2/neu transgenic (HER2‐Tg) mouse model is classically considered a prototype for HER2‐positive breast cancer, its gene expression profile in tumors resembles that of the luminal subtypes,^[^ ^46^ ^]^ and tumoral ERα in this strain was activated by DEHP (Figure S1G, Supporting Information). In this strain, DEHP shortened the time to tumor onset (Figure 1D), reduced tumor‐free survival (Figure 1E), and increased the growth rate of mammary tumors in vivo (Figure 1F). Furthermore, tumors from DEHP‐exposed HER2‐Tg mice showed a higher organoid formation capability compared to those from control mice (Figure 1G). This observation was corroborated by ex vivo treatment with DEHP, which resulted in an enlargement of tumor organoids derived from control tumors of HER2‐Tg mice (Figure S1H, Supporting Information). These findings indicated that DEHP exposure significantly contributes to the early initiation and progression of breast cancer. Therefore, the DEHP exposure was further utilized as a model to explore mechanisms underlying the early onset of breast cancer.

### 2.2 DEHP Exposure Enriches Cancer Stem Cell Populations

**Figure 2:**
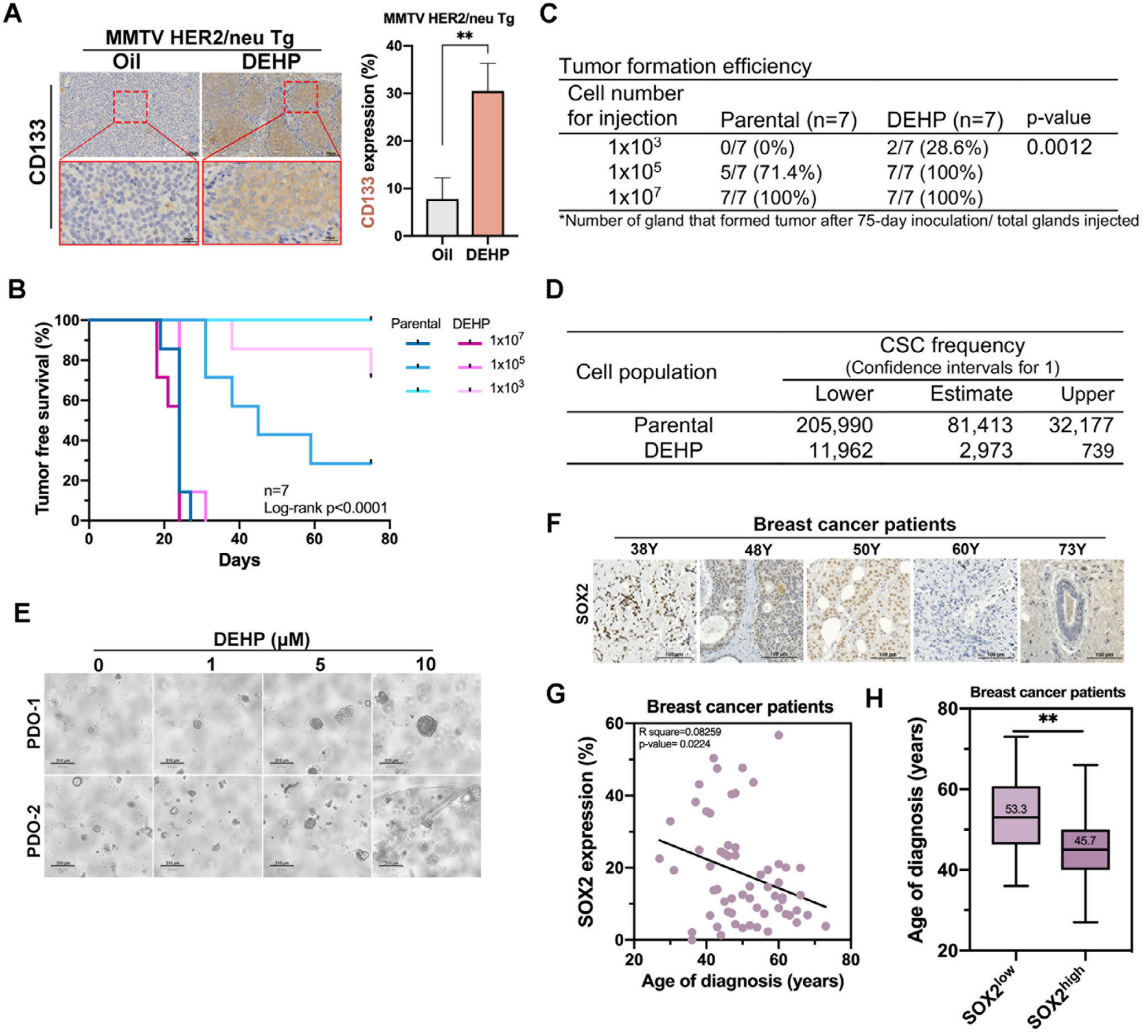
DEHP promotes the cancer stemness of breast cancer in vivo. A) CD133 expression in the tumors of HER2‐Tg mice from Figure 1D was detected in IHC analysis. *n =* 5 per group. B–D) In the limiting dilution assay, the tumor‐free survival rate (B), tumorigenicity (C), and cancer stem cell frequency (D) in NOD‐SCID mice with T47D xenograft tumors (*n*  = 7 in each group) were shown. A *p*‐value of the tumor‐free survival rate was calculated by using the log‐rank (Mantel‐Cox) test. E) Treatments with DEHP for 7 days increased the formation of organoids from tumors of ER‐positive breast cancer patients (PDO‐1 and PDO‐2). F–H) The SOX2 expression in tumor tissues determined in IHC analysis (F) negatively correlated with the age of diagnosis in breast cancer patients (G). The age of diagnosis was younger in patients with high SOX2 expression (H). Data were shown as the mean ± SD. ^∗∗^ *p* < 0.01 versus the control group, Student's *t*‐test.

Tumor initiation capability is attributed to higher cancer stemness activity.^[^ ^47^ ^]^ The result of Gene Set Enrichment Analysis (GSEA) revealed significant enrichment in the regulation of stem cell proliferation in luminal type MCF7 breast cancer cells (Figure S2A, Supporting Information) in response to DEHP 10 µm, a concentration similar to the highest tolerable daily intake in humans. Moreover, the expressions of cancer stemness markers,^[^ ^48^ ^]^ including CD133 (**Figure** **2**A) as well as SOX2 and ALDH1 (Figure S2B, Supporting Information), in mouse tumor tissues were elevated in response to chronic DEHP exposure in immunohistochemical (IHC) staining and Western blot analysis. We further explored whether DEHP promotes cancer stemness by assessing the spheroid formation of MCF7 cells. The short‐term treatment with DEHP for 7 days dose‐dependently increased the number of spheroids (Figure S2C, Supporting Information), without affecting the cell proliferation even at higher concentrations up to 100 µm (Figure S2D,E, Supporting Information). To investigate the impact of long‐term DEHP exposure on cancer stemness, MCF7 cells were treated with DEHP at 1 µm (DEHP1) and 10 µm (DEHP10) for over a month. Chronic DEHP treatment notably stimulated the formation of spheroids (Figure S2F, Supporting Information), accompanied by a slight induction of cell growth (Figure S2G, Supporting Information). Furthermore, DEHP1 and DEHP10 MCF7 cells expressed higher cancer stem cell markers, including SOX2 expression at both protein (Figure S2H, Supporting Information) and mRNA (Figure S2I, Supporting Information) levels and ALDH activity (Figure S2J, Supporting Information). Interestingly, DEHP10 cells also showed nuclear expression of SOX2 within the spheroid structure (Figure S2K, Supporting Information).

To confirm the contribution of DEHP to cancer stemness in vivo, the tumorigenicity of serially diluted parental and DEHP10 of luminal T47D breast cancer cells injected into the mammary gland fat pad in NOD‐SCID mice was examined. This limited dilution assay revealed that mice implanted with DEHP10 T47D cells showed a shorter tumor‐free survival rate (Figure 2B). After implantation with 1 × 10^3^ cells for 75 days, 28.6% of the mice bearing DEHP10 cells developed tumors, while none of the mice bearing parental T47D cells formed tumors (Figure 2C). The CSC frequency in extreme limiting dilution analysis (ELDA)^[^ ^49^ ^]^ showed 43.5‐fold higher in the DEHP‐exposed group (1/739) than in the parental group (1/32 177) (Figure 2D). In addition, the patient‐derived organoids (PDO) were induced by DEHP in a dose‐dependent manner (Figure 2E). Moreover, the expression of SOX2 is inversely correlated with the age at breast cancer diagnosis (Figure 2F,G, and Table S1, Supporting Information), and presents a 3.76‐fold odds ratio (OR) for the early onset of breast cancer (Table S2, Supporting Information). The average diagnosis age of breast cancer patients with high SOX2 expression (SOX2^high^) is younger than that of patients with low SOX2 expression (SOX2^low^) (Figure 2H).

CSCs present a tough challenge in elimination due to their inherent resistance to anti‐cancer drugs.^[^ ^50^ ^]^ Notably, overexpression of BCRP, an efflux transporter that actively expels various anti‐cancer drugs from the cells, has been widely recognized for conferring chemoresistance in CSCs.^[^ ^51^ ^]^ The protein (Figure S3A, Supporting Information), mRNA (Figure S3B, Supporting Information), and Hoechst33342 efflux activity (Figure S3C, Supporting Information) of BCRP in MCF7 cells were induced by DEHP. Furthermore, the DEHP‐exposed MCF7 cells increased resistance to the chemotherapeutic agent doxorubicin, a known substrate of BCRP, in both cell growth (Figure S3D, Supporting Information) and clonogenic formation (Figure S3E, Supporting Information) assays. These findings indicate that DEHP enhances breast cancer stemness, emphasizing its potential contribution to not only the early onset but also the chemoresistance of breast cancer.

### 2.3 DEHP‐Enhanced Glutaminolysis Metabolism Upregulates Mitochondrial Respiratory Capacity

**Figure 3:**
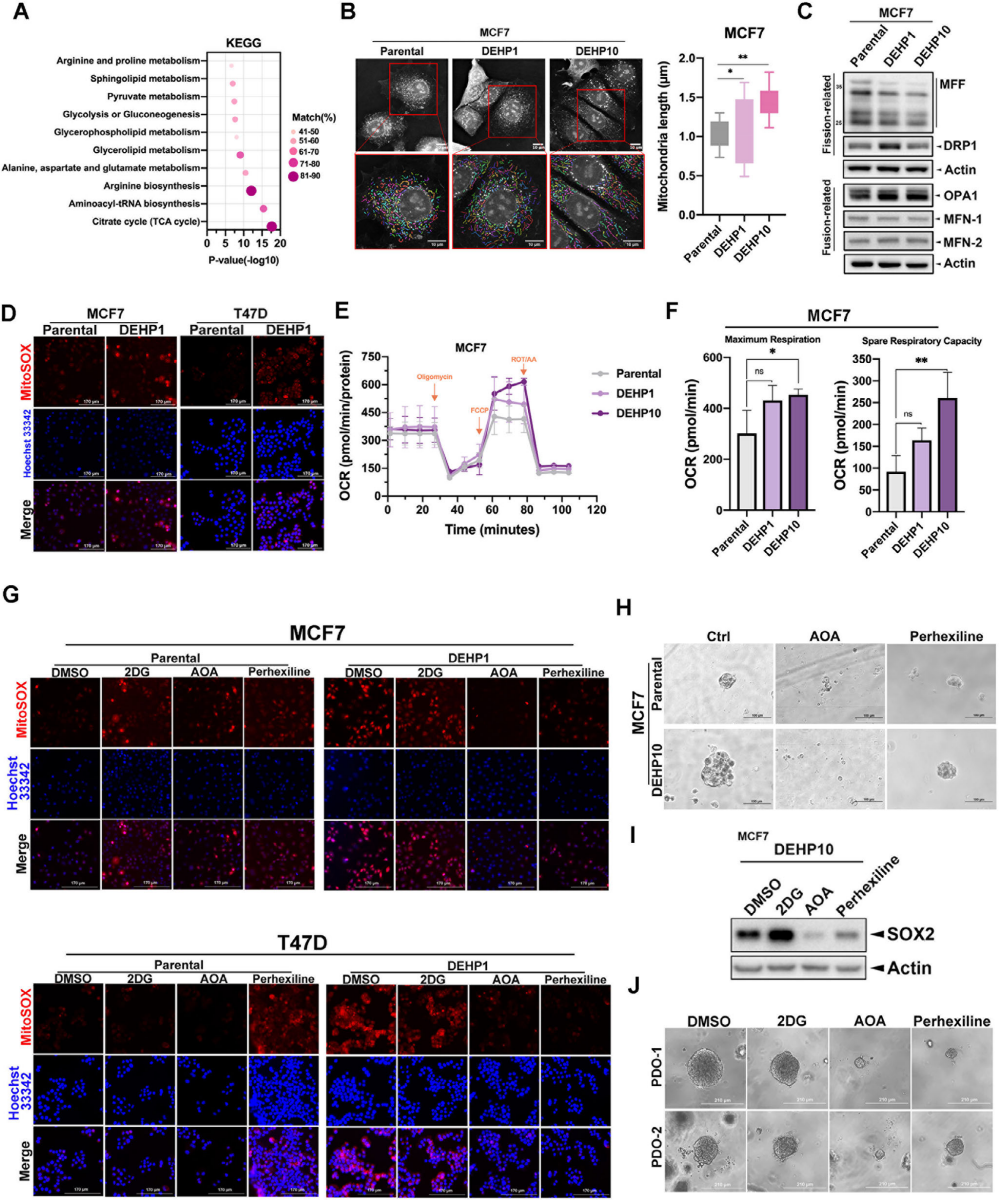
DEHP induced mitochondrial respiration capacity. A) The enriched metabolic pathways were identified with joint metabolomics and transcriptomics analysis by the Genes and Metabolites (GAM) web service. B) The tomographic image and average total length of mitochondria in parental and DEHP10 of MCF7 cells were captured with the 3D Cell Explorer‐fluo and were quantified with the Smart Mitochondrial Assay^LIVE^ (Nanolive). C) The protein expressions of mitochondria dynamics‐related genes in parental and DEHP‐exposed MCF7 cells were determined in a Western blot with the indicated antibodies. D) Staining of parental cells and DEHP1 of MCF7 and T47D cells with MitoSOX Red was visualized by fluorescence microscopy. E and F) The OCR in parental cells and DEHP‐exposed MCF7 cells was analyzed by Seahorse Metabolic Analyzer following the sequential addition of mitochondrial inhibitors, oligomycin, FCCP, rotenone/antimycin A in (E). Maximal respiration and spare respiratory capacity were calculated in (F). G) Staining of parental and DEHP1 of MCF7 and T47D cells with MitoSOX Red following the 24‐hr treatments with AOA (200 µm), Perhexiline (5 µm), and 2DG (25 µm) was visualized by fluorescence microscopy. H) The growth of parental and DEHP10 of MCF7 cells cultured in matrigel was suppressed by AOA (200 µm) or Perhexiline (5 µm) for 7 days. I) MCF7/DEHP10 was treated with 2DG (25 µm), AOA (200 µm), and Perhexiline (5 µm) for 48 h, and SOX2 expression was examined in Western blot analysis. J) The growth of patient‐derived organoids (PDO‐1 and PDO‐2) was suppressed by AOA (200 µm) and Perhexiline (5 µm) but not 2DG (25 µm) for 7 days. Images represent one representative experiment from three independent experiments. Data were shown as the mean ± SD. ns. not significant; ^∗^ *p* < 0.05; ^∗∗^ *p* < 0.01 versus the control group. Student's *t*‐test (B) and one‐way ANOVA with Tukey's multiple comparisons test (F).

To explore the molecular mechanisms underlying DEHP‐enhanced cancer stemness, we examined the alteration of DEHP‐associated gene expression profile in RNA‐sequencing analysis. The data from the Gene Ontology (GO) annotation enrichment analysis revealed the upregulation of numerous gene sets associated with mitochondrial respiration (Figure S4A–C, Supporting Information). A subsequent joint pathway analysis of metabolomic and transcriptomic data revealed that enriched metabolism pathways, particularly the citrate cycle, were elicited by DEHP (**Figure** **3**A). Cancer stem cells have been demonstrated to rely more on mitochondrial respiration for energy generation compared to general cancer cells in various cancer types.^[^ ^52^ ^]^ Elevated mitochondrial fusion activity enables the stem‐like properties of CSCs.^[^ ^53^, ^54^ ^]^ Interestingly, DEHP increased the length of mitochondria to form a mitochondrial network (Figure 3B) accompanied by a reduction of MFF protein (Figure 3C) but not mRNA (Figure S4D, Supporting Information) level without affecting the protein level of other mitochondrial proteins (Figure S4E, Supporting Information), suggesting that DEHP shifts the balance of mitochondria dynamic toward fusion by suppressing the protein abundance of MFF, a fission factor. In support of the enhancement of mitochondrial fusion, DEHP treatment also increased mitochondrial reactive oxygen species (ROS) levels in breast cancer cell lines stained with MitoSOX (Figure 3D), suggesting that mitochondrial fusion was augmented by DEHP to increase mitochondrial respiration. Indeed, DEHP induced the oxygen consumption rate (OCR) (Figure 3E), especially the maximal mitochondrial respiration and spare respiratory capacity (Figure 3F), in MCF7 cells. Inhibition of glutamine metabolism by targeting aminotransferase with aminooxyacetate (AOA) or fatty acid β‐oxidation by targeting carnitine palmitoyltransferase (CPT) with perhexiline, but not glycolysis by targeting hexokinase 2 with 2‐deoxy‐D‐glucose (2DG), primarily suppressed the maximal mitochondrial respiration and spare respiratory capacity (Figure S4F, Supporting Information) and mitochondrial ROS production (Figure 3G) in DEHP‐exposed cells. Moreover, AOA and perhexiline suppressed DEHP‐induced spheroid formation (Figure 3H) and SOX2 expression (Figure 3I) in breast cancer cell lines. Similar suppressive effects of AOA and perhexiline on the formation of PDO (Figure 3J) were also observed. These results suggest that DEHP‐induced cancer stemness involves mitochondria fusion and subsequent metabolic reprogramming of glutamine and fatty acids.^[^ ^55^ ^]^

### 2.4 DEHP‐Induced Glutamine Uptake Enhances the Tricarboxylic Acid (TCA) Cycle and Nucleotide Synthesis via Oxidative Phosphorylation

**Figure 4:**
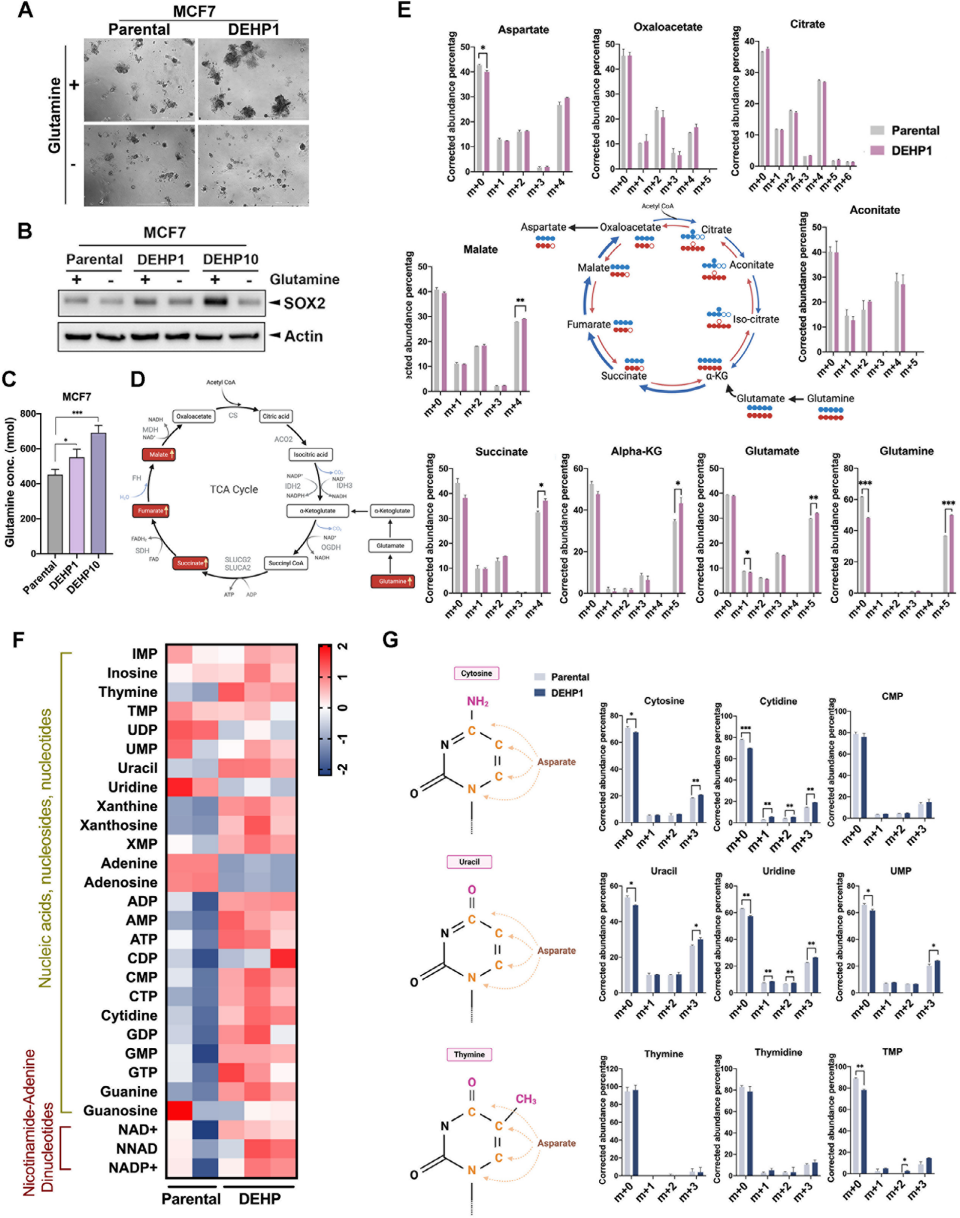
Glutamine is essential for DEHP‐induced cancer stemness. A) The spheroid formation was higher in DEHP1 than in parental MCF7 cells and was reduced by glutamine removal from the culture medium. Images represent one representative experiment from three independent experiments. B) SOX2 expression was increased in DEHP1 and DEHP10 of MCF7 cells and was repressed by glutamine removal in Western blot analysis. C) The glutamine level in cell lysates harvested from DEHP‐exposed cells was measured in colorimetric assays. *n =* 3 per group. D) The schematic illustration of the DEHP‐upregulated TCA cycle metabolites (labeled in red) in mass spectrum analysis. E) Metabolic flow analysis tracing with[U‐^13^C_5_] glutamine showed that DEHP enhanced glutaminolysis through oxidative TCA cycle (blue) but not reductive carboxylation (red). *n =* 2 per group. F) Nucleotide‐related metabolites in parental and DEHP1 MCF7 cells were analyzed by mass spectrometry. G) The DEHP‐enhanced nucleotides generated from[U‐^13^C_5_] glutamine were determined in metabolic flow analysis. *n =* 2 per group. (E and G). m+n, where m is the mass of the unlabeled metabolite and n denotes the number of 13C‐labeled carbon atoms. Data were shown as the mean ± SD. ^∗^ *p* < 0.05; ^∗∗^ *p* < 0.01; ^∗∗∗^ *p* < 0.001 versus the control group, one‐way ANOVA with Tukey's multiple comparisons test (C) and Student's *t*‐test (E and G). The illustration was created with Biorender.com.

Supporting a role of glutaminolysis in DEHP‐induced cancer stemness, glutamine deprivation produced a markedly greater reduction in sphere formation (**Figure** **4**A) and SOX2 expression (Figure 4B) in DEHP‐treated MCF7 cells than in parental cells. These findings indicate that DEHP reprograms glutamine metabolism, rendering cells more dependent on glutaminolysis to sustain stemness features. In parallel to these findings, DEHP exposure increased the intracellular levels of glutamine in colorimetric assays (Figure 4C). Interestingly, DEHP‐exposed MCF7 cells also showed an increase in the intracellular levels of TCA cycle metabolites, including succinate, fumarate, and L‐malate (Figure 4D and Figure S4G, Supporting Information), suggesting that DEHP‐driven glutamine uptake may enhance TCA cycle activity. To investigate whether the increase in intracellular glutamine level resulted from enhanced uptake or endogenous synthesis, cells with glutamine deprivation for 3 h were reintroduced with glutamine. The highest intracellular glutamine level was observed at the fourth hour and was enhanced by DEHP (Figure S4H, Supporting Information). These findings suggest that DEHP enhances glutamine uptake to increase mitochondrial oxidative phosphorylation.

Next, the ^13^C metabolic flux analysis (MFA) with[U‐^13^C_5_] glutamine tracer was performed to further quantify the glutamine‐derived metabolic alterations in response to DEHP exposure. The results showed that fractional enrichments of m+5 glutamine, glutamate, and α‐ketoglutarate (αKG), along with m+4 succinate and malate, were increased by DEHP (Figure 4E). This suggests that DEHP induced glutamine uptake to promote the oxidative TCA cycle metabolism. The[U‐^13^C_5_] glutamine metabolic flow was increased until m+4 oxaloacetate toward aspartate, but not citrate and aconitate (Figure 4E). Since aspartate provides a source of carbon and nitrogen for pyrimidine synthesis,^[^ ^56^ ^]^ the glutamine metabolic flow may promote the de novo nucleotide. Indeed, most nucleotides were increased in the DEHP‐exposed MCF7 cells in metabolomic mass spectrum analysis (Figure 4F). The results of MFA with[U‐^13^C_5_] glutamine tracer further demonstrated the enhancement of glutamine‐driven nucleotide biosynthesis, particularly cytosine, uracil, and their derivatives, by DEHP exposure (Figure 4G). These findings suggest that DEHP enhances the oxidative TCA cycle metabolism and de novo nucleotide biosynthesis by increasing glutamine uptake.

### 2.5 SLC6A14 Upregulation Contributes to DEHP‐Induced Glutamine Uptake and Cancer Stemness

**Figure 5:**
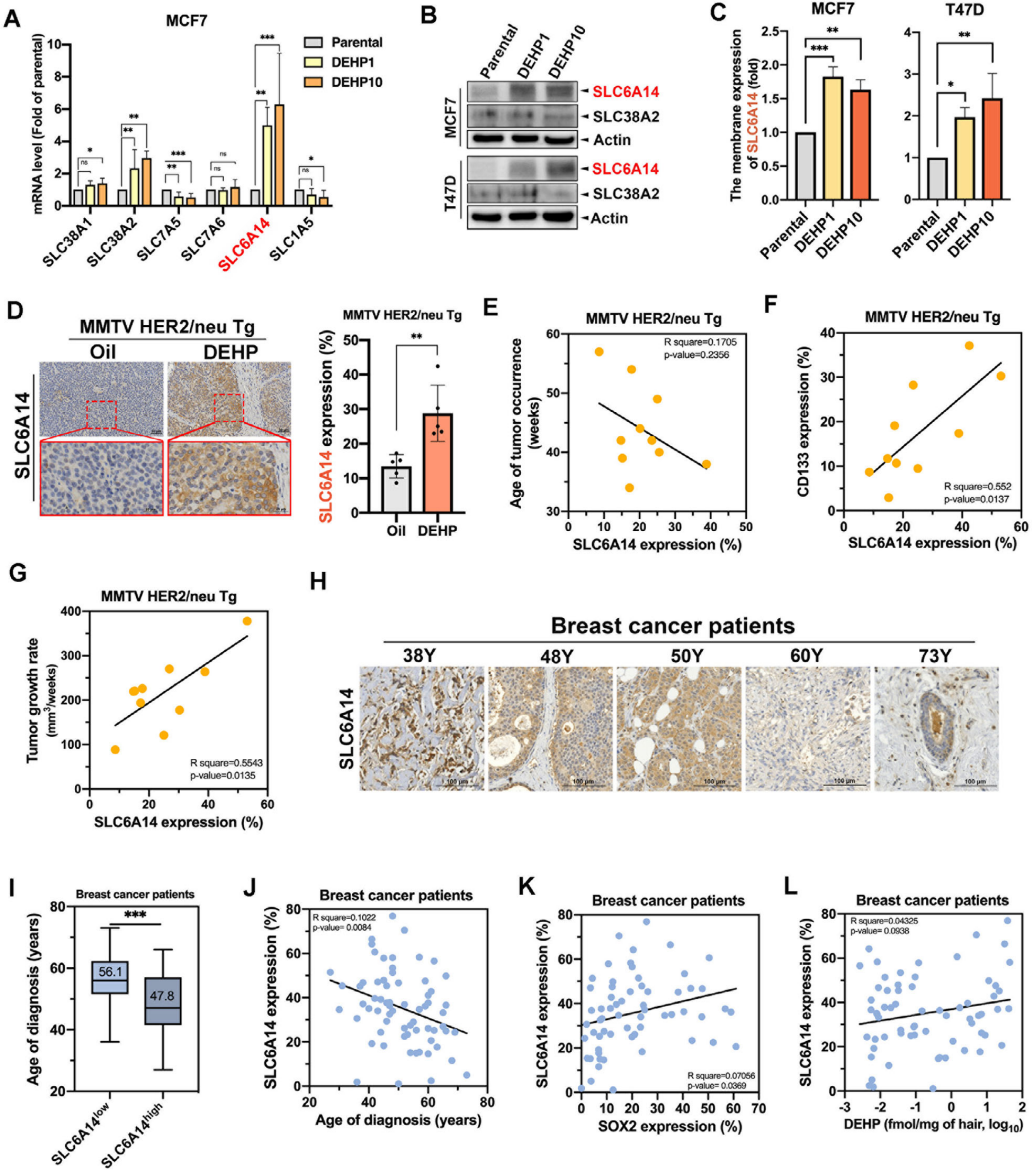
Upregulation of SLC6A14 by DEHP is associated with early‐onset and cancer stemness of breast cancer. A) The changes in mRNA levels of various glutamine transporters in MCF7 cells in response to DEHP were examined in RT‐qPCR analysis. The mRNA levels of target genes were normalized to actin. *n =* 3 per group. B) Protein expressions of SLC6A14 and SLC38A2 in parental and DEHP‐exposed MCF7 and T47D cells were determined in Western blot with the indicated antibodies. C) The expression of SLC6A14 on the cell membrane of parental and DEHP‐exposed MCF7 and T47D cells was analyzed by flow cytometry. *n =* 3 per group. D–G) The SLC6A14 expression in tumors of HER2‐Tg mice from Figure 1D was examined in IHC staining (D). *n =* 6 per group. The correlations of SLC6A14 with the age of tumor occurrence (E), CD133 expression (F), and the rate of tumor growth (G) in HER2‐Tg mice were further analyzed. *n =* 10. H) SLC6A14 in breast carcinoma was higher in younger patients. I) The age of diagnosis was younger in patients with high SLC6A14 expression. J) Negative correlation between the age of diagnosis age and tumorous SLC6A14 protein expression in human breast cancer patients. K,L) The correlation of SLC6A14 expression with SOX2 expression (K) or hair level of DEHP (L) in breast cancer patients. Data were shown as the mean ± SD. ns. not significant; ^∗^ *p* < 0.05; ^∗∗^ *p* < 0.01; ^∗∗∗^ *p* < 0.001 versus the control group, one‐way ANOVA with Tukey's multiple comparisons test (A and C) and Student's *t*‐test (D and I).

Among various glutamine transporters, solute carrier family 6 member 14 (SLC6A14) was then screened out as the potential transporter in mediating DEHP‐facilitated glutamine uptake due to its upregulation by DEHP exposure in RNA‐sequencing analysis (Figure S5A, Supporting Information). The significant upregulation of SLC6A14 mRNA (**Figure** **5**A) and protein (Figure 5B) in DEHP‐exposed MCF7 cells was further validated by RT‐qPCR analysis and Western blot analysis, respectively. The increase in the membrane protein level of SLC6A14, but not SCL38A2, by DEHP was further observed in flow cytometry analysis (Figure 5C and Figure S5B, Supporting Information). The expression of SLC6A14 was also induced by DEHP in a dose‐dependent manner in mouse breast tumors (Figure 5D). Furthermore, SLC6A14 expression was higher in tumors occurring at a younger age (Figure 5E) and positively correlated with the expression of CD133 (Figure 5F) and the rate of tumor growth (Figure 5G) in HER2‐Tg mice. The expression of SLC6A14 in tumor tissues was higher in younger breast cancer patients (Figure 5H,I, and Table S3, Supporting Information) with an odds ratio of 3.896 (p‐value = 0.025) (Table S4, Supporting Information) and negatively correlated with the age at diagnosis (Figure 5J). The expression of SLC6A14 also exhibited a positive correlation with the level of SOX2 in tumors with an odds ratio of 4.40 (p‐value = 0.012) (Figure 5K and Table S5, Supporting Information) and the hair level of DEHP in breast cancer patients (Figure 5L). These clinical findings support that SLC6A14 upregulation by DEHP may contribute to cancer stemness and be associated with the early onset of breast cancers.

### 2.6 ERα Mediates DEHP‐Induced SLC6A14 Expression

Plasticizers exhibit a structural and functional resemblance to estrogen and are considered environmental hormones.^[^ ^57^ ^]^ Previous studies have reported the binding affinity of DEHP to estrogen receptor alpha (ERα).^[^ ^58^ ^]^ In HER2‐Tg mice, the expression of pERα is positively correlated with SLC6A14 level (Figure S6A, Supporting Information). We next examined the involvement of ERα in DEHP‐induced SLC6A14 expression, and the results demonstrated that the DEHP‐induced SLC6A14 expression was dose‐dependently suppressed by fulvestrant, a selective estrogen receptor degrader (SERD) (Figure S6B, Supporting Information) and by ERα shRNA (Figure S6C, Supporting Information) in both MCF7 and T47D cell lines. The inhibitory effect of fulvestrant on DEHP‐induced SLC6A14 mRNA levels was also observed (Figure S6D, Supporting Information). These results collectively suggest that DEHP transcriptionally upregulates SLC6A14 expression, at least partially, through the activation of ERα. Phthalates share endocrine‐disrupting pathways but exhibit quantitative differences in receptor engagement and downstream activation.^[^ ^59^ ^]^ Consistent with this, docking analysis predicts stronger ERα binding for DEHP than for representative phthalate metabolites (Figure S6E, Supporting Information), and only DEHP elicited a robust SLC6A14 induction under our assay conditions (Figure S6F, Supporting Information). These observations support a model in which preferential ERα engagement by DEHP contributes to SLC6A14 upregulation, while not excluding contributions from other receptors or co‐factors.

### 2.7 Targeting SLC6A14 Suppresses DEHP‐Associated Cancer Stemness and Tumor Progression

**Figure 6:**
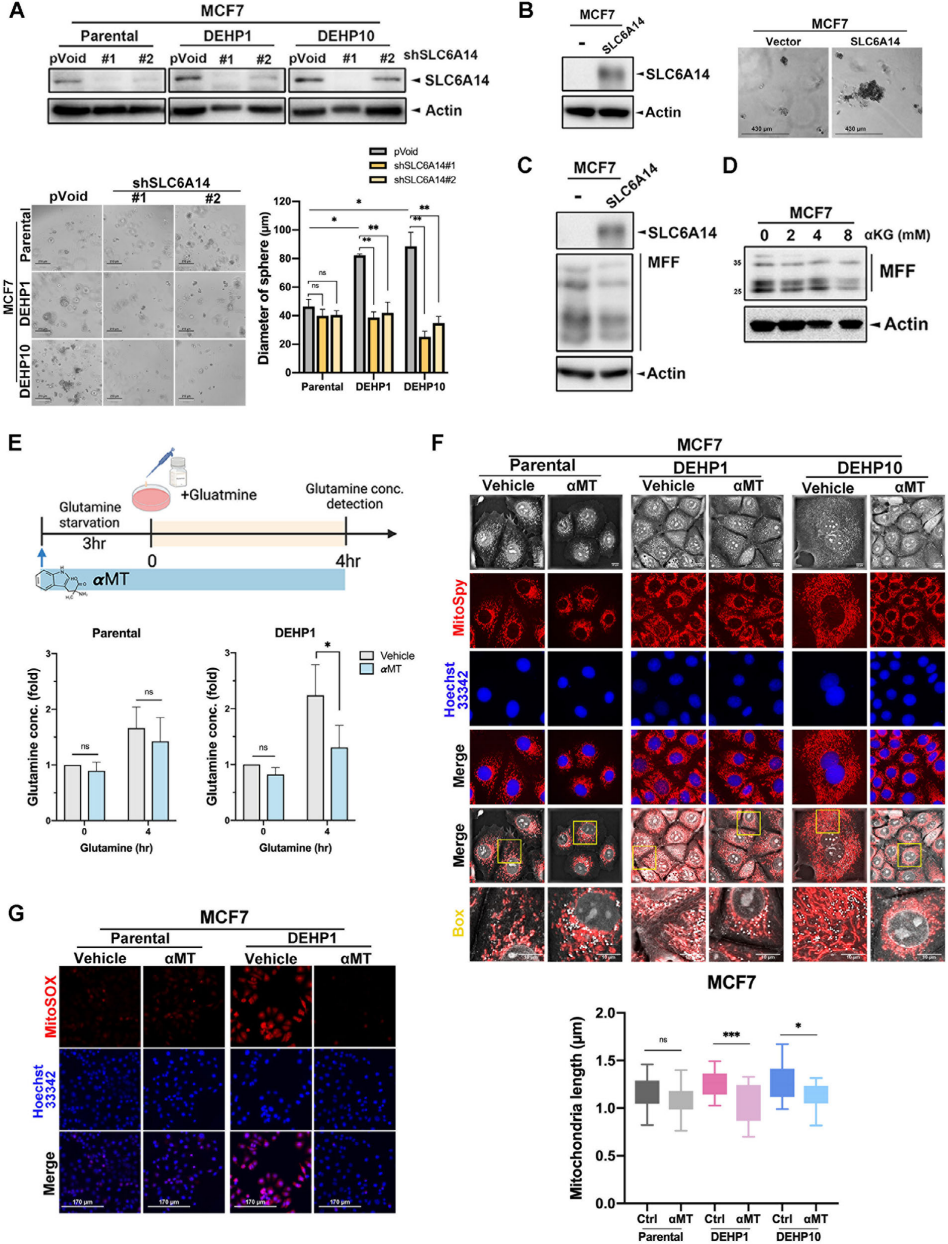
Glutamine transport activity of SLC6A14 is required for mitochondria fusion. A) Parental and DEHP‐exposed MCF7 cells were transfected with SLC6A14 shRNA and cultured in an ultra‐low dish for 7 days, followed by spheroid formation assays. B) Spheroid formation of SLC6A14‐overexpressed MCF7 cells was measured in a 3D cell culture system. C) MCF7 cells were transfected with SLC6A14 plasmids. The expression of MFF was determined in Western blot analysis. D) MCF7 cells treated with αKG for 48 h were subjected to Western blot analysis of MFF expression. E) Parental and DEHP1 MCF7 cells were pretreated with 2.5 mm αMT in a glutamine‐depleted medium for 3 h and then supplemented with 2 mm glutamine for 4 h. The cell lysates were harvested and subjected to glutamine concentration analysis. *n =* 3. F,G) Parental and DEHP1 MCF7 cells were treated with 2.5 mm αMT for 48 h followed by staining with MitoSpy (F) and mitochondrial superoxide indicator MitoSOX Red (G). Images represent one representative experiment from three independent experiments. Data were shown as the mean ± SD. ns, not significant; ^∗^ *p* < 0.05; ^∗∗^ *p* < 0.01; ^∗∗∗^ *p* < 0.001 versus the control group, one‐way ANOVA with Tukey's multiple comparisons test (A) and Student's *t*‐test (E).

In support of the critical role of SLC6A14 in DEHP‐induced glutamine uptake and cancer stemness, DEHP‐promoted spheroid formation of MCF7 cells was significantly suppressed by silencing SLC6A14 expression with specific shRNA (**Figure** **6**A). Conversely, overexpression of SLC6A14 in MCF7 cells significantly increased spheroid formation (Figure 6B). These findings indicate that SLC6A14 plays a crucial role in promoting cancer stemness. Moreover, silencing SLC6A14 decreased DEHP‐enhanced mitochondrial fusion in MCF7 cells (Figure S7, Supporting Information). In contrast, SLC6A14 overexpression (Figure 6C) and αKG treatment (Figure 6D) repressed the protein level of MFF. Pretreatment with a SLC6A14 inhibitor α‐methyltryptophan (αMT) suppressed glutamine uptake (Figure 6E). Moreover, DEHP‐promoted mitochondrial fusion (Figure 6F) and ROS production (Figure 6G) were suppressed by αMT in MCF7 cells. It suggested that SLC6A14‐dependent glutamine uptake mediates DEHP‐elicited mitochondria fusion and respiration by suppressing MFF expression to promote cancer stemness.

**Figure 7:**
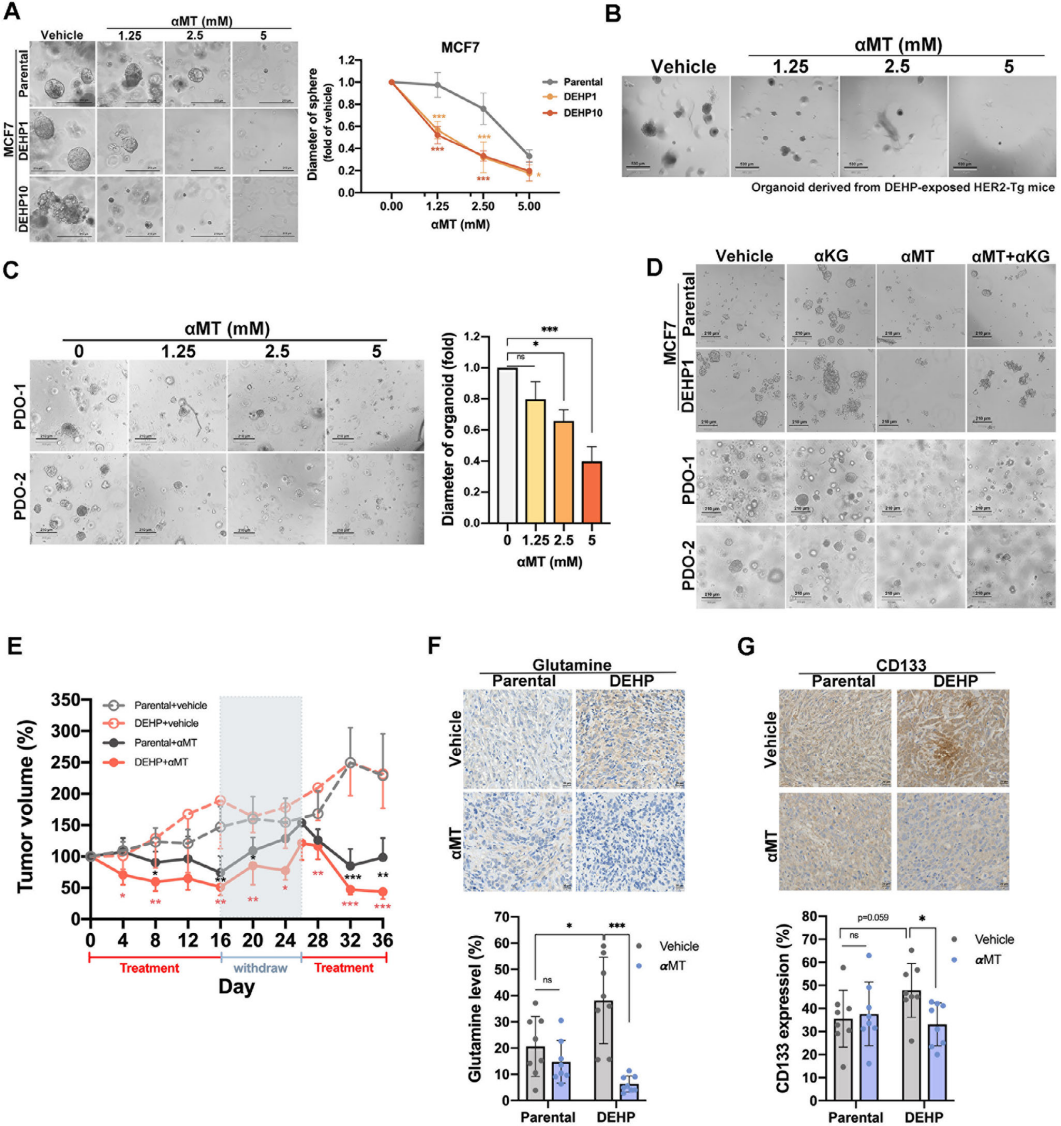
SLC6A14 is a potential therapeutic target for DEHP‐induced cancer stemness. A) Spheroid formation of parental cells and DEHP‐exposed MCF7 cells treated with αMT (1.25, 2.5, and 5 mm) for 7 days was measured in a 3D cell culture system. B,C) The organoid formation of DEHP‐exposed HER2‐Tg tumors (B) and patient‐derived organoid (PDO‐1 and PDO‐2) (C) was dose‐dependently suppressed by αMT for 7 days. D) Co‐treatment with αKG 4 mm prevents the suppression of organoid formation by αMT 2.5 mm. E–G) Mice with parental and DEHP10 T47D xenograft tumors were treated with αMT as indicated. The tumor size was measured in (E), and the representative histological images and quantitative results of glutamine level (F) and CD133 (G) in tumors were shown. *n* = 8 per group. Images represent one representative experiment from three independent experiments. Data was shown as the mean ± SD. ns. not significant; ^∗^ *p* < 0.05; ^∗∗^ *p* < 0.01; ^∗∗∗^ *p* < 0.001 versus the control group, one‐way ANOVA with Tukey's multiple comparisons test (A and C) and Student's *t*‐test (E–G).

The size of DEHP‐induced spheroids was more significantly vulnerable to αMT (**Figure** **7**A) but not to SLC38A2 inhibitor α‐methylaminoisobutyric acid (MeAIB) (Figure S8A, Supporting Information). Similarly, treatment with αMT (Figure 7B), but not MeAIB (Figure S8B, Supporting Information), significantly decreased the size of organoids derived from DEHP‐treated HER2‐Tg mice. Consistently, organoids derived from two breast cancer patients (PDO‐1 and PDO‐2) were sensitive to αMT (Figure 7C). In support of the contribution of SLC6A14‐mediated glutamine metabolism to cancer stemness, its downstream metabolite αKG rescued the sphere and organoid formation of MCF7 cells and PDO from the suppression by αMT (Figure 7D). Importantly, SLC6A14 inhibition by αMT dramatically suppressed the growth of the xenograft tumors from the T47D DEHP clone in NOD‐SCID mice (Figure 7E). IHC staining revealed that αMT decreased the level of glutamine (Figure 7F), concurrently suppressing the expression of CD133 in DEHP‐exposed tumors (Figure 7G).

**Figure 8:**
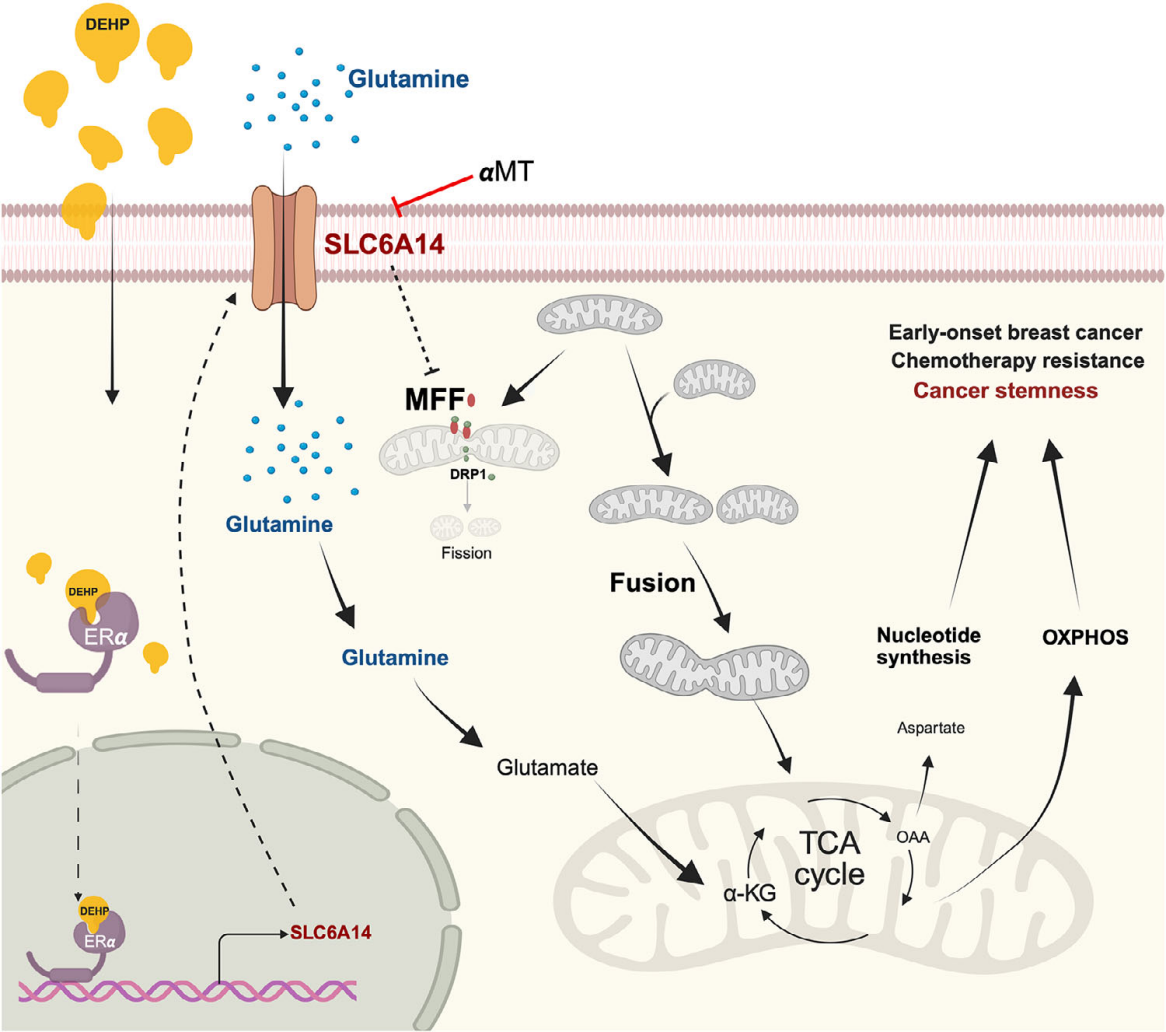
DEHP exposure promotes cancer stemness and early onset of breast cancer via mitochondrial fusion and glutamine metabolism by upregulating SLC6A14. In response to DEHP exposure, SLC6A14 was upregulated in an ERα‐dependent manner to facilitate glutamine uptake and mitochondrial fusion via MFF suppression. The enhanced glutamine metabolism fuels oxidative phosphorylation and de novo nucleotide biosynthesis to promote cancer stemness, resulting in the early onset and chemoresistance of breast cancer. Targeting SLC6A14 glutamine transporter activity was suggested as a promising therapeutic strategy for EOBC patients.

Furthermore, higher SLC6A14 expression was associated with non‐response to chemotherapeutic agents in the ROC plotter (Figure S9A, Supporting Information). SLC6A14 inhibition with αMT sensitized DEHP‐exposed MCF7 cells to doxorubicin (Figure S9B, Supporting Information). These findings collectively suggest that the SLC6A14‐enhanced glutamine uptake, mitochondrial fusion, and subsequent glutaminolysis contribute to DEHP‐induced TCA cycle activity and cancer stemness, resulting in the development of early‐onset breast cancer (**Figure** **8**). Therefore, SLC6A14 is a potential target to improve the therapeutic outcome for EOBC patients.

## 3 Discussion

Other than genetic deficiency, exposure to an environmentally hazardous plasticizer DEHP was demonstrated to contribute to the early onset of breast cancer through augmentation of cancer stemness relying on SLC6A14‐dependent mitochondria dynamics in this study. SLC6A14 has been suggested as a potential target for several solid tumors, including pancreatic, cervical, and colon cancers.^[^ ^60^ ^]^ The high SLC6A14 level was also significantly correlated with poor post‐progress survival of breast cancer in a Kaplan‐Meier analysis.^[^ ^61^ ^]^ Our study revealed the promoting effects of DEHP‐activated ERα on SLC6A14 upregulation and cancer stemness. Given that a substantial fraction of luminal B cancers are HER2‐positive (≈41%), our findings in the ERα‐responsive MMTV‐HER2/neu mice model support the plausibility of a DEHP‐facilitated early‐onset trajectory within both luminal A and B diseases. Indeed, the higher expressions of SLC6A14 (Figure S10A, Supporting Information) and SOX2 (Figure S10B, Supporting Information) in tumor tissues and younger age of diagnosis (Figure S10C, Supporting Information) were observed in patients with the ERα‐positive breast cancers, particularly in luminal B subtype, further substantiating the significance of the ERα/SLC6A14 axis in influencing cancer stem cell properties for the early onset of breast cancer in response to DEHP exposure.

CSCs employ unique metabolic pathways for self‐renewal and chemoresistance, enabling the identification of promising therapeutic targets with fewer potential adverse effects.^[^ ^48^, ^62^ ^]^ Targeting glutamine metabolism has shown promise in circumventing chemoresistance of breast cancer^[^ ^63^ ^]^ and ovarian cancer,^[^ ^64^ ^]^ implying the addiction of glutamine in the self‐renewal of CSC in many cancer types. By utilizing glutamine as the primary metabolic source, CSCs rely largely on mitochondrial OXPHOS activity to produce the principal energy through the TCA cycle.^[^ ^65^ ^]^ Beyond energy generation, αKG‐derived citrate contributes to lipid and de novo membrane synthesis.^[^ ^66^ ^]^ Additionally, glutamine and αKG‐derived aspartate also function as nitrogen and carbon donors for pyrimidine synthesis.^[^ ^67^ ^]^ Increased synthesis of both pyrimidine^[^ ^68^ ^]^ and lipid synthesis^[^ ^48^ ^]^ is believed to be crucial for the maintenance of cancer stemness.

To meet the high metabolic demand, CSCs exhibit a branched mitochondria network to efficiently supply bioenergetic substances.^[^ ^69^ ^]^ Exposure to DEHP increases this interconnected mitochondrial structure by shifting the mitochondrial dynamics toward fusion by reducing MFF expression in a SLC6A14‐dependent manner. Consistently, glutamine is required for the suppression of mitochondrial fission by reducing the mTOR/MFF/DRP1 pathway.^[^ ^70^ ^]^ However, DEHP exposure did not alter MFF transcripts (Figure S4D, Supporting Information), suggesting post‐transcriptional regulation. Glutamine‐driven anaplerosis expands mitochondrial acyl‐CoA pools^[^ ^71^ ^]^ and nutrient‐responsive lysine acylations in modulating the polyubiquitination, proteasomal turnover, and function of proteins.^[^ ^72^, ^73^, ^74^ ^]^ Overexpression of SLC6A14 and increased α‐KG flux also reduced MFF protein abundance (Figure 6C,D). It is worth further addressing whether SLC6A14‐mediated glutamine uptake influences MFF protein stability via acylation‐dependent mechanisms.

Competition for glutamine uptake exists between cancer cells and immune cells in the tumor microenvironment (TME).^[^ ^75^ ^]^ In glutaminase‐deficient mouse tumor models, restricted glutamine utilization in cancer cells increases the glutamine availability to tumor‐infiltrated T lymphocytes for their antitumor activity.^[^ ^76^ ^]^ Targeting glutamine transporters, including SLC1A5, SLC38A1, SLC38A2, and SLC6A14, has been proposed as a potential therapeutic strategy for cancer.^[^ ^77^ ^]^ However, the upregulation of SLC1A5, SLC38A1, or SLC38A2 to meet the bioenergetic and biosynthetic demands has also been linked to the activation and proliferation of cytotoxic T cells.^[^ ^77^, ^78^ ^]^ Therefore, exploiting selective CSC dependency on SLC6A14‐dependent glutamine supply as a metabolic vulnerability would be a promising anti‐tumor strategy for EOBC patients with less negative impact on T cell activation. Glutamine‐addicted tumor cells competitively consume glutamine to indirectly polarize macrophages to the pro‐tumorigenic M2 subtype.^[^ ^79^ ^]^ Interestingly, SLC6A14 expression in tumour tissues has been found to be positively correlated with tumour‐associated M2 macrophages.^[^ ^61^ ^]^ Therefore, selectively targeting SLC6A14 in tumor cells may not only inhibit the self‐renewal of CSCs but also alleviate glutamine competition to repolarize macrophages back to the anti‐tumorigenic M1 subtype in the TME.

Belonging to the SLC6 family, SLC6A14 is known as an amino acid transporter B^0,+^ (ATB^0,+^) with a broad spectrum of substrates, including glutamine and other neutral cationic amino acids.^[^ ^80^ ^]^ The dramatic rescue effect of αKG, the downstream metabolite of glutamine, on the αMT‐elicited sphere and organoid inhibition (Figure 7D) indicates that SLC6A14 contributes to DEHP‐mediated cancer stemness mainly relying on its glutamine uptake activity. Although our data with SLC6A14 genetic perturbation and uptake‐rescue controls support an on‐target mechanism for αMT, multiple studies show that αMT is a substrate of LAT1 (SLC7A5)^[^ ^81^, ^82^ ^]^ and may engage other neutral amino acid transport systems. Consequently, αMT‐induced phenotypes can reflect composite effects across transports, and off‐target actions cannot be excluded. The development and application of more selective SLC6A14 inhibitors would reduce off‐target liabilities and improve translational potential.

Although our findings suggest a correlation between elevated hair DEHP levels and EOBC, the relatively limited cohort size in this study may not provide sufficient statistical power for definitive epidemiological conclusions. Potential confounding factors,^[^ ^83^, ^84^, ^85^, ^86^, ^87^ ^]^ such as socioeconomic status, diet, hormonal status, lifestyle, and co‐exposures to other environmental pollutants, were not fully controlled for and cannot be excluded from influencing the observed associations. Therefore, these results underscore the need for larger and well‐controlled epidemiological studies to validate the link between DEHP exposure and EOBC risk.

## 4 Conclusion

In conclusion, our study demonstrated that exposure to DEHP enhances breast cancer stemness, potentially playing a role in the development of early‐onset breast cancer. Mechanistically, DEHP induces the upregulation of SLC6A14 to promote glutaminolysis, mitochondrial respiratory capacity, and de novo DNA synthesis involving glutamine uptake and mitochondrial fusion. By supplying bioenergetic substances, the upregulated SLC6A14 plays a key role in augmenting the self‐renewal ability of CSCs. These findings explore a metabolic mechanism underlying plasticizer‐associated early‐onset breast cancer. This study further suggests that the first FDA‐approved SLC6A14 inhibitor is worthy of development as a new therapeutic strategy for patients with EOBC, who often face significant challenges due to chemoresistance and aggressive tumor behavior.

## 5 Experimental Section

### Cell Lines and Cell Culture

Human breast cancer MCF7 (RRID: CVCL_0031), T47D (RRID: CVCL_0553), and MDA‐MB‐231 (RRID: CVCL_0062) cell lines were purchased from the American Type Culture Collection (ATCC). The DEHP‐exposed clones of MCF7 and T47D cell lines were established by long‐term treatment with DEHP for over 1 month. They were maintained in the medium with 1 or 10 µm DEHP and named as DEHP1 and DEHP10, respectively. The concentration range (1–10 µm) of DEHP used in vitro was selected based on previous reports demonstrating its biologically relevant effects on breast epithelial and cancer cells.^[^ ^88^, ^89^ ^]^ All of the cell lines were grown in Dulbecco's modified Eagle's medium/F12 medium supplemented with 10% fetal bovine serum, 100 units mL^−1^ penicillin, and 100 µg mL^−1^ streptomycin with 95% air and 5% CO_2_ at 37 °C.

### Clinical Data and Tissue Specimens

This study analyzed clinical data from 105 breast cancer patients who received therapies at Chung Shan Medical University Hospital (Taichung, Taiwan) between 2018 and 2019. Among these, residual breast tumor tissue samples were available for further analysis in 67 cases. All patients provided written informed consent, and the study protocol (CS1‐22107) was approved by the Institutional Research Board Committee. The inclusion criteria encompassed individuals with histologically confirmed invasive ductal carcinoma, with samples representing a range of breast cancer molecular subtypes and tumor grades. Tissue collection was performed randomly, without selection bias, to ensure representative distribution across subtypes.

A normal cohort of 22 people attending routine health examinations was enrolled. Eligibility required no personal history of malignancy or breast disease; individuals who were pregnant/lactating or reported recent acute illness were excluded. Hair samples were collected using a standardized protocol for DEHP biomonitoring. This normal cohort served as the reference group for determining baseline hair concentrations of DEHP in healthy individuals.

### DEHP Extraction from Human Hair

As described previously,^[^ ^21^ ^]^ two separate bundles of hair from hair root to end per patient were collected and cleaned with sterilized distillation‐distillation H_2_O two times. After drying, the hair will be cut into small pieces and weighed. The hair bundles (25 mg), cut from the root and collected into glass bottles, were incubated with 1 mL of methanol/TFA (8.5:1.5) overnight to extract DEHP metabolites. The extracted solution was collected in new glass bottles and dried in a chemical hood. The dried residues were dissolved with 30% methanol and analyzed by a liquid chromatography‐tandem mass spectrometry (LC‐MS/MS) system. The average level of DEHP metabolites from the hair of 22 healthy volunteers was measured and used as the basal control for normalization.

### The Examination of ADH in Mouse Mammary Glands

Four‐week‐old female ICR mice (RRID: IMSR_CRL:022) were divided into normal diet and high‐fat diet groups (45 kcal% fat) to establish the obesity model. Each group of mice was fed with corn oil or DEHP at doses of 0.21 and 1.73 mg kg^−1^ daily for one year.

Microscopic examination of mouse mammary tissues stained with hematoxylin and eosin (H&E) revealed distinctive features such as cuboidal or polygonal morphology and columnar hyperchromatic cells, characterized by a high nucleus/cytoplasm (N/C) ratio within the ductal epithelium (Figure S1E, Supporting Information). These morphological alterations were consistent with the definition of ADH,^[^ ^90^ ^]^ a recognized precancerous lesion that substantially elevates the risk of developing breast ductal carcinoma by four to five times.^[^ ^90^ ^]^ The myoepithelium markers cytokeratin 5/6 (CK5/6)^[^ ^91^ ^]^ and p63^[^ ^92^ ^]^ were employed to discern hyperplasia of mammary ductal epithelial cells.

### The Effect of DEHP on Tumor Progression in the HER2‐Tg Mouse Model

Four‐week‐old female MMTV‐HER2/neu transgenic mice (RRID: IMSR_JAX:0 02376) were fed with corn oil or DEHP at doses of 0.21 mg kg^−1^ daily. Tumor initiation and growth were measured using a caliper and calculated as 1/2 x length x width^2^. The experiment was concluded for each mouse when its tumor reached a diameter of over 20 mm.

### Tumor Initiation Assays in a Subcutaneous Xenograft Mouse Model

Prior to the day of cell injection, female NOD‐SCID mice (RRID: IMSR_CRL:394) were anesthetized and subcutaneously implanted with an E2 pellet. Parental and DEHP10 T47D cells at densities of 1 × 10^7^ cells were then collected and mixed with Matrigel Matrix, followed by subcutaneous injection into the left and right mammary fat pads, respectively. Tumor initiation and growth were measured using a caliper and calculated as 1/2 x length x width^2^.

### Limiting Dilution Assay

To quantify CSC frequency, an in vivo limiting‐dilution assay (LDA) was performed. Parental and DEHP10 T47D cells, diluted to densities of 1 × 10^3^, 1 × 10^5^, and 1 × 10^7^ per 100 µL, were injected into the left and right mammary glands of NOD‐SCID mice, respectively. Tumor formation was monitored over the course of˙75 days, and the tumor‐initiating/CSC frequency and 95% confidence intervals were estimated using Extreme Limiting Dilution Analysis (ELDA; http://bioinf.wehi.edu.au/software/elda; RRID: SCR_01 8933), and group differences were evaluated by the χ^2^ likelihood‐ratio test.^[^ ^49^ ^]^

### Mouse Model Experiments

All mice were obtained from accredited animal facilities and maintained under specific pathogen‐free (SPF) conditions. All animals were acclimated for one week before experimental procedures. Animals were allocated to control and treatment groups using simple randomization. Animals in each group were housed and treated under consistent environmental conditions to minimize bias. Daily treatments (oral DEHP or αMT) were administered for the designated period, and measurements were taken weekly or on a specified day.

Throughout the experimental procedures, measures were taken to minimize animal pain and distress. Isoflurane anesthesia was used for all subcutaneous injections. Humane endpoints were established prior to the study. Animals were monitored daily for signs of discomfort, including reduced activity, abnormal posture, weight loss, or tumors exceeding 20 mm in diameter. Mice were euthanized if any of these standards were happened. No unexpected adverse events were observed during the study. Mice treated with DEHP or fed high‐fat diets did not exhibit overt signs of toxicity, and all experimental animals survived to the study endpoint. The outcome assessments were not blinded to the treatment groups. No specific inclusion or exclusion criteria were established a priori for the animal samples. All animals and samples that met the general experimental protocol were included in the analysis. No animals or data points were excluded from the analysis in any of the experimental groups. Normality and homogeneity of variance were not formally tested before statistical comparisons. Data were assumed to follow a normal distribution based on previous studies using similar models.

All animal experiments in this study were approved by the Institutional Animal Care and Use Committee (IACUC) of China Medical University (Approval Number: CMUIACUC‐2021‐103) and followed NIH guidelines.

### Western Blot Analysis

Cells were washed with ice‐cold PBS, and lysates were collected in RIPA buffer (50 mm Tris, 150 mm NaCl, 10 mm EDTA, 1% Triton X‐100, 0.1% SDS) containing complement and protease inhibitors. After sonication and centrifugation at 15 000 rpm for 15 min, the supernatant of cell lysates was collected, and protein concentration was measured by using Bradford protein assay (Bio‐Rad). After protein separation with SDS‐PAGE and transfer to PVDF membrane (0.45 µm, GE Healthcare), the protein‐transferred membrane was blotted with 5% milk in TBST buffer for 1 h, followed by incubation with the indicated primary antibodies and HRP‐labeled secondary antibodies. The expression level of target proteins was catalyzed with ECL reagent (GE Healthcare or Millipore) and was determined by detecting the chemoluminescence signal. Antibodies against SOX2 (Santa Cruz; sc‐365823; RRID: AB_10 842 165), CD133 (abcam; ab19898;; RRID: AB_470 302), ERα (Santa Cruz; sc‐8002; RRID: AB_627 558), pERα (Ser108; Cell Signaling; 2511; RRID: AB_331 289), SLC6A14 (Thermo Fisher; PA5‐42452; RRID: AB_2 576 522), SLC38A2 (Medical&Biological Laboratories (MBL); BMP081; RRID: AB_10 597 880), BCRP (Millipore; MAB4146; RRID: AB_2 220 429), mitochondrial dynamics antibody sampler kit II (Cell Signaling; 74 792), mitochondrial marker antibody sampler kit (Cell Signaling; 8674; RRID: AB_11 217 817), β‐actin (Sigma‐Aldrich; A2228; RRID: AB_476 697) and α‐Tubulin (Sigma‐Aldrich; T5168; RRID: AB_477 579) were purchased commercially.

### Reverse‐Transcription (RT) and Quantitative Real‐Time Polymerase Chain Reaction (qPCR)

The mRNA level of the target gene was determined in the RT‐qPCR reaction. The RT reaction in 20 µL includes 1 µg of total RNA, 10 mm of Oligo‐dT, 10 mm dNTP, and DEPC H_2_O. The mixture was heated to 65 °C for 5 min followed by the addition of 4 µL of 5X NNLV buffer, 10 mm DTT, and 1 µL MMLV transcriptase. For qPCR reaction, 1 µL of the product from RT reaction was added with SYBR Green Master Mix and a pair of primers. The target gene was amplified for 45 cycles using the following conditions, including denaturation at 95 °C for 10 s, annealing at 55 °C for 20 s, and extension at 72 °C for 1 s.

### Spheroid Formation Assay

For spheroid formation, both ultra‐low attachment spheroid cultures and Matrigel‐based 3D systems have been utilized to investigate CSC properties. The ultra‐low attachment spheroid assay enriches for cells with anchorage‐independent growth and anoikis resistance, which were characteristic of CSC populations. Matrigel‐based 3D cultures provide an extracellular matrix environment that promotes cell–matrix interactions in tissues. These features were also critical for maintaining CSC phenotypes. Therefore, both culture conditions serve as platforms to evaluate CSC.^[^ ^93^ ^]^ Breast cancer cells were cultured in ultra‐low attachment plates or on the Matrigel‐coated plates at a density of 1 × 10^4^ cells per well. In ultra‐low attachment plates, cells were cultured with Dulbecco's modified Eagle's medium/F12 medium containing 1x B27 Supplement (Gibco#1 774 010), 20 ng mL^−1^ basic fibroblast growth factor, 20 ng mL^−1^ epidermal growth factor, and 4 µg mL^−1^ insulin. On the plates with 100% Matrigel in the bottom and medium containing 2% Matrigel, cells were cultured for 7 days. The numbers and size of the spheroids were measured and quantified by using ImageJ software.

### Tumor‐Derived Organoid Culture

Breast tumor specimens were obtained from mice or surgical resections under institutional ethical approval and patient consent. Fresh tumor tissues were rinsed in PBS, and transferred into ice‐cold ADF+++ medium. In a sterile culture hood, tissues were minced into 1–2 mm^3^ fragments and incubated with 1 mg mL^−1^ collagenase (Sigma, C9407) in ADF+++ medium at 37 °C for 1 h with gentle agitation. The digested cell suspension was passed through a 70 µm cell strainer. If red blood cell (RBC) contamination was observed, RBC lysis buffer (Roche, 11 814 389 001) was applied for 5 min at room temperature and stopped by adding ADF+++ medium. Cells were pelleted by centrifugation at 500 × g for 5 min and resuspended in ADF+++ medium. Cell mixed with basement membrane extract (BME) was used to prepare domes (30–40 µL) in 24‐well plates. After polymerization at 37 °C for 15 min, 400 µL of breast cancer organoid medium was added. Medium was changed every 4 days or as needed based on organoid growth.

The breast organoid culture medium was based on ADF+++ (Advanced DMEM/F12 (Gibco,12 634 028) supplemented with 1% GlutaMAX (Gibco, 35 050 061), 10 mm HEPES, 1× Primocin (InvivoGen, ant‐pm‐05), and 1% penicillin‐streptomycin) and included the following components: 50% L‐WRN conditioned media (generated in our laboratory), 5 nM Neuregulin‐1 (Peprotech, 100–03), 5 ng mL^−1^ human FGF7 (hFGF7, Peprotech, AF100‐19), 20 ng mL^−1^ human FGF10 (hFGF10, Peprotech, AF100‐26), 5 ng mL^−1^ human EGF (hEGF, Peprotech, AF‐100‐15), 0.5 µm A83‐01 (Tocris, 2939), 5 µm Y‐27632 (AbMole, M1817), 0.5 µm SB202190 (Sigma, S7067), 1× B‐27 (Gibco, 17504–44), 1.25 mm N‐acetylcysteine (Sigma, A9165), 5 mm Nicotinamide (Sigma, N0636). For mouse‐derived mammary tumor organoids, the medium was identical except that hFGF10 and hEGF were excluded, and 50 ng mL^−1^ mouse EGF (mEGF; Peprotech, 315‐09) was used.

All components were sterile filtered and stored as aliquots at −80 °C. Medium was freshly prepared weekly due to the short half‐life of several supplements.

### ALDH Activity Assay

The cells were trypsinized and washed with PBS. The ALDEFLUOR kit (STEM CELL #17K84807) was applied to analyze the population with ALDH activity. The cells were incubated in an assay buffer with an ALDH substrate and with and without a specific ALDH inhibitor, diethylaminobenzaldehyde (DEAB), at 37 °C for 30 min, followed by Flow Cytometry analysis.

### BCRP Activity Assay

To assess the functional activity of BCRP, the accumulation of Hoechst33342 was measured, a cell‐permeable hydrophobic substrate for BCRP that fluoresces upon binding with DNA in cells.^[^ ^94^ ^]^ Cells were incubated overnight in a complete growth medium at 37 °C. After being washed with PBS, cells were stained with 1 µm bisBenzimide Hoechst33342 trihydrochloride (Sigma) in phenol red‐free medium at 37 °C for 4 h.

### Colony Formation Assay

Cells were seeded at a density of 1 × 10^4^ cells in a 6‐well plate and treated with DEHP and Doxorubicin for a week. The colony‐forming cells were stained with 1 m crystal violet for 30 min followed by dissolving in 30% ethanol.

### RNA‐Seq Analysis

Total RNA extracted from parental or DEHP‐exposed MCF7 cells using Trizol reagent was subjected to library generation by using the KAPA mRNA HyperPrep Kit (KAPA Biosystems, Roche, Basel, Switzerland) following the manufacturer's guidelines with the addition of index codes for sample attribution. Library sequencing quality was further confirmed using the Qubit@ 2.0 Fluorometer (Thermo Scientific) and Agilent Bioanalyzer 2100 system. The final step of sequencing the library was conducted on an Illumina NovaSeq6000 platform, generating 150 bp paired‐end reads. Differential expression was analyzed with DESeq2 using raw counts, size‐factor normalization, and dispersion modeling. Wald tests (two‐sided) were performed for specified contrasts, and *P* values were adjusted by the Benjamini–Hochberg procedure to control the false discovery rate (FDR); genes with FDR < 0.05 were considered significant.

### Metabolomics Analysis

Parental and DEHP‐exposed MCF7 cells (2 × 10^6^ cells) were lysed with 200 µL ddH_2_O, and the supernatant was collected after centrifuging at 15000 rpm for 10 min. The supernatant was further mixed with four times the volume of 100% methanol and centrifuged at 15000 rpm for 10 min. The supernatant was collected and lyophilized using the Eppendorf Vacufuge Concentrator to obtain the metabolites. Metabolite profiling was subsequently analyzed by LC‐ESI‐MS analysis, which consisted of an ultra‐performance liquid chromatography (UPLC) system (ACQUITY UPLC I‐Class, Waters) and an ESI/APCI source of a 4 kDa quadrupole time‐of‐flight (TOF) mass spectrometer (Waters VION, Waters). The flow rate was set 0.2 mL min^−1^ with column temperature at 35 °C. Separation was performed with reversed‐phase liquid chromatography (RPLC) on a BEH C18 column (2.1×100 mm, Walters) with 5 µL sample injection. The elution started from 99% mobile phase A (ultrapure water + 0.1% formic acid) and 1% mobile phase B (100% methanol + 0.1% formic acid), held at 1% B for 0.5 min, raised to 90% B in 5.5 min, held at 90% B for 1 min, and then lowered to 1% B in 1 min. The column was equilibrated by pumping 1% B for 4 min. Data for fatty acids and organic acids and data for DEPH were acquired by ESI‐ mode and ESI+ mode, respectively, under the following conditions: capillary voltage of 2.5 kV, source temperature of 100 °C, desolvation temperature at 250 °C, cone gas maintained at 10 L h^−1^, desolvation gas maintained at 600 L h^−1^, and MS^E^ mode with a range of m/z 100–1000 and 0.5 s scan time. The acquired data were processed by UNIFI software (Waters) with an illustrated chromatogram and summarized in an integrated area of signals.

### Determination of Mitochondrial Superoxide

To examine the effect of DEHP on mitochondrial superoxide production, both parental and DEHP‐exposed MCF7 cells were cultured under specific experimental conditions. The MCF7 cells were incubated with 5 µm of MitoSOX Red (Invitrogen; M36008) at 37 °C for 20 min and hoechest33342 for 5 min. The live cells were then visualized using the Revolve Fluorescence Microscope (ECHO).

### Mitochondria Morphology Analysis

Live cells cultured in µ‐Dish^35mm, low^ (Ibidi) cells were stained with 50 nm MitoSpy (BioLegend, 424 807) for 20 min and were imaged on a 3D Cell Explorer‐fluo (Nanolive; digital holo‐tomographic microscopy) equipped with a stage‐top incubator (37 °C, 5% CO_2_). For each field, a 3D refractive‐index (RI) stack was acquired under identical exposure and reconstruction settings across conditions. Raw RI volumes were reconstructed in Steve software and exported as 16‐bit TIFF stacks.

For morphometric analysis, label‐free 3D holotomographic images were acquired using a Nanolive 3D Cell Explorer. The resulting 3D RI stacks were processed and compressed into maximum intensity projections (MIPs) for subsequent 2D analysis. Segmentation and quantification of individual mitochondria were performed on the EVE Explorer platform (Nanolive) using the EVE Analytics module and its dedicated Smart Mitochondrial Assay^LIVE^ pipeline. This automated process involved the segmentation of mitochondria from the cellular background, followed by the quantification of various morphological parameters. The primary readout for the analysis was the mean total length of each mitochondrion. At least 10–20 cells per condition were analyzed from ≥3 independent experiments, with 2 randomly selected fields.

### Metabolic Flux Analysis (MFA)

MCF7 cells, incubated with a culture medium containing 25 mm glucose, 2 mm [U‐^13^C_5_] glutamine, and 10% dialyzed FBS for 12 h, were lysed with the cool‐extracted solvent (acetonitrile: methanol: water = 2:2:1). Following sonication in a cold bath and centrifugation at 13000 rpm for 10 min, the supernatant was collected and dried using the Eppendorf Vacufuge Concentrator to obtain the metabolites.

The samples were analyzed on an Agilent 1290 II Infinity Ultra‐High‐Performance Liquid Chromatography system (Agilent Technologies, Palo Alto, CA, USA) with an Agilent 6545XT quadrupole time‐of‐flight (Q‐TOF) mass spectrometer (Agilent Technologies, Palo Alto, CA, USA). The sample was separated by using an ACQUITY UPLC BEH amide column (1.7 µm, 2.1 × 100 mm, Waters Corp., Milford, MA, USA) at 40 °C. The mobile phases were composed of deionized water (eluent A) and LC‐MS grade 90% acetonitrile (v/v) (eluent B), both eluents with 15 mm ammonium acetate and 0.3% NH_3_∙H_2_O. The flow rate was 300 µL min^−1^, and the injection volume of the sample was 5 µL. The mass spectrometer was equipped with an Agilent Jet‐stream source operating in positive and negative full‐scan ion mode, collected from m/z of 60–1500. The chromatogram acquisition, detection of mass spectral peaks, and their waveform processing were performed using Agilent LC/MS Data Acquisition software 9.0 and Agilent Qualitative Analysis 10.0 (Agilent, USA). Isotopologue extraction was performed, and natural isotope abundance was corrected using Agilent Profinder 10.0 (Agilent, USA). The mass tolerance was set to ±10 ppm, and retention time tolerance was ±0.6 min in isotopologue extraction parameters.

### Flow Cytometry Assay

Cells with the indicated treatments were trypsinized and washed with PBS. After fixation with 4% formaldehyde for 15 min at 4 °C, the cells were stained with anti‐SLC6A14 (MBL; BMP052; RRID: AB_1 953 038) and SLC38A2 (MBL; BMP081; RRID: AB_10 597 880) diluted in blocking buffer (PBS containing 0.5% BSA) at room temperature for 1 h. Following washing with PBS, cells were incubated with fluorescein‐conjugated anti‐rabbit antibody (Invitrogen; Alexa Fluor 647; RRID: AB_2 535 812) diluted in blocking buffer in the dark for 30 min at room temperature. After removing the excessive antibodies, the cells were re‐suspended in fresh PBS, and 1 × 10^4^ cells were analyzed by Flow Cytometry.

### Immunohistochemistry Staining

The paraffin‐embedded tissue sections were deparaffinized in xylene and rehydrated in alcohol, sequentially. To prevent endogenous peroxidase activity, tissue slides were immersed in 3% hydrogen peroxide. After antigen retrieval in citrate buffer by using a pressure cooker for 10 min, the slides were immunostained with the primary antibody at 4 °C overnight and incubated with polymer HRP‐conjugated secondary antibody. The tissue slides were then stained with DAB solution and hematoxylin. The antibodies used in IHC staining include anti‐p63 (abcam; ab124762; RRID: AB_10 971 840), anti‐CK5/6 (Thermo Fisher; MA5‐12429; RRID: AB_10 982 609), anti‐CD133 (abcam; ab19898; RRID: AB_470 302), anti‐pERα (Ser108; Bioss; bs‐3130R; RRID: AB_10 856 911), anti‐SLC6A14(abcam; ab254786; RRID: AB_3 073 883), and anti‐SOX2(Santa Cruz, sc‐365823; RRID: AB_10 842 165), anti‐glutamine (abcam; ab9445; RRID: AB_307 259) antibodies.

### Measurement of Oxygen Consumption Rate (OCR)

Breast cancer cells were seeded in a Seahorse XF24 cell culture plate at 0.2 × 10^5^ per well. Before 1 h of the assay, the cell culture medium was replaced with XF Base Medium with glutamine 2 mm and glucose 5 mm. During OCR acquisitions in Seahorse XF24 Analyzer (Agilent), 1 µm oligomycin, 1 µm Carbonyl cyanide‐p‐trifluoromethoxyphenylhydrazone (FCCP), and 1 µm rotenone and Antimycin A (Rot*/*AA) were sequentially added to the reaction.

### ROC Plotter Analysis

The receiver operating characteristic (ROC) Plotter (http://www.rocplot.org), a tool integrating gene expression and therapy response data from the transcriptome of breast cancer patients,^[^ ^95^ ^]^ was used to predict the expression of SLC6A14 in ER‐positive breast cancer patients in response to chemotherapy by setting 219 795_at as the probe, relapse‐free survival at 5 years and any chemotherapy for treatment, and ER‐positive status.

### Statistical Analysis

Statistical analyses were performed using Student's *t*‐test for comparisons between two groups. For comparisons among more than two groups with a single independent variable, one‐way ANOVA followed by Tukey's post hoc test was used. For experiments involving two independent variables, a two‐way ANOVA followed by Tukey's post hoc test was used to assess the main effects and interactions. Data were expressed as the mean ± SD, with a sample size of n ≥ 3. *P*‐values were calculated using two‐tailed tests, with *p* < 0.05 considered statistically significant. The significance of tumor‐free survival was evaluated using the log‐rank (Mantel–Cox) test. Pearson's chi‐square test was applied to analyze categorical variables in mice and breast cancer patient characteristics, and unconditional logistic regression was used to calculate odds ratios.

## Conflict of Interest

The authors declare no conflict of interest.

## Author Contributions

D.‐W. H. and C.‐H. H. contributed equally to this work. D.W.H. and W.C.H. did Conceptualization. D.W.H. and W.C.H. did Methodology. D.W.H., Y.H.H., Y.L.W., S.W.H., F.J.C., T.K.H, B.R.C., B.W.W., L.C.K, and D.Y.L. did Investigation. D.W.H. did Writing‐Original Draft. W.C.H. M.C.H., and Y.‐H.H. did Writing‐Review & Editing. D.W.H. did Visualization. C.H.H., W.C.H., and S.Y.W. did Funding Acquisition. C.H.H., M. H.Y., Y.J.C, and L.C.L. did Resources. W.C.H. did Supervision.

## Supporting information



Supporting Information

## Data Availability

The data that support the findings of this study are available from the corresponding author upon reasonable request.
