# Supplementary material for: SLC6A14 Drives Mitochondrial Fusion and Oxidative Phosphorylation to Promote Cancer Stemness and Early‐Onset of Breast Cancer
Source: Adv Sci (Weinh). 2025 Sep 17;12(45):e10811. doi: 10.1002/advs.202510811 (PMC12677700; doi:10.1002/advs.202510811)
Supplement: Supplementary file 1 — Supporting Information [file ADVS-12-e10811-s001.docx]

Supporting Information

SLC6A14 Drives Mitochondrial Fusion and Oxidative Phosphorylation to Promote Cancer Stemness and Early-Onset of Breast Cancer.

Dai-Wei Hu, Chih-Hao Huang, Yu-Hao He, Ya-Ling Wei, Shu-Wei Hu, Fang-Ju Cheng, Thanh Kieu Huynh, Bo-Rong Chen, Bo-Wei Wang, Li-Chi Kuan, Der-Yen Lee, Ming-Hsin Yeh, Ya-Jen Chang, Liang-Chih Liu, Mien-Chie Hung, and Wei-Chien Huang^*^

**

**

**Figure S1. DEHP exposure contributes to the initiation and progression of early-onset breast cancer.** The breast cancer diagnosed age ≤ 45 years old was defined as EOBC. The hair level DEHP over 10-fold of the average level in healthy donors (2.3 fmol/mg) was defined as DEHP-positive. (A-C) The distribution of breast cancer cases by age (A), breast cancer subtype (B), and hair DEHP level (C). (D) The proportion of EOBC and non-EOBC cases among DEHP-positive (left, n = 15) and DEHP-negative (right, n = 90) breast cancer patients. (E) The distribution of DEHP levels in hair (presented in Z score) by age. (F) ICR mice were assigned to groups with a normal diet (ND) or high-fat diet (HFD, 45 kcal% fat) receiving daily doses of oil, low (0.21 mg/kg), or high DEHP (1.73 mg/kg) for a year. The mammary gland tissues were subjected to whole-mount staining with hematoxylin and eosin, CK5/6, and P63. (G) Tumors of HER2-Tg mice from Fig. 1D were stained with pERα antibody by IHC and the nuclear level of pERα was quantitated. *n=*5 per group. (H) Organoid formation of oil-treated HER2-Tg mice tumor was enhanced by exposure to DEHP for 10 days. Data were shown as the mean ± SD. ∗*p* < 0.05; ∗∗*p* < 0.01 versus the control group, Student’s t-test.





**Figure S2. Plasticizer DEHP induced cancer stemness in ER-positive breast cancer cells.** (A) The gene set involved in the regulation of stem cell proliferation in MCF7 cells was increased by DEHP in GSEA analysis. (B) Total tumor lysates of HER2-Tg mice from Fig. 1D were prepared and subjected to western blot analysis for SOX2, ALDH, and CD133 expressions. The changes in these protein levels were quantified and normalized to actin. (C) The spheroid formation of MCF7 cells was increased by DEHP exposure for 7 days. *n*=3. (D and E) MCF7 cells were treated with low dose (1, 5, and 10 μM) (D) or high dose (50 and 100 μM) (E) of DEHP for 3 days, followed by the proliferation assay with Incucyte Live-Cell analyzer. (F) DEHP-exposed MCF7 cells were cultured in a three-dimensional culture system followed by a spheroid formation assay. The diameter of the spheres was measured and quantified. *n*=3. (G) The proliferation rate of DEHP-exposed MCF7 cells was slightly higher than that of their parental cells in Incucyte Live-Cell analysis. (H and I) The protein (H) and mRNA (I) expressions of SOX2 were increased in DEHP-exposed MCF7 cells in Western blot and RT-qPCR analyses, respectively. *n*=3. (J) The percentage of ALDH-positive populations was higher in DEHP1 than in parental MCF7 cells in ALDEFLUOR assays. *n*=3. (K) Total expression and nuclear localization of SOX2 were higher in the spheroids of DEHP10 than in parental MCF7 cells. Images represent one representative experiment from three independent experiments. Data were shown as the mean ± SD. ∗p < 0.05; ∗∗p < 0.01 versus the control group, Student’s t-test (B, F, I, and J) and one-way ANOVA with Tukey’s multiple comparisons test (C).





**Figure S3. DEHP promotes chemoresistance by inducing the expression of BCRP.** (A and B) BCRP protein (A) and mRNA (B) expressions were higher in DEHP-exposed MCF7 cells in Western blot and in RT-qPCR analyses, respectively. *n*=3. (C) Parental and DEHP-exposed MCF7 cells were stained with Hoechst 33342 dye for 4 hours, and the dye exclusion was measured and quantified by Image J. *n*=3. (D and E) Parental and DEHP-exposed MCF7 cells were treated with the indicated concentrations of doxorubicin. The cell numbers were counted every 6 hours in IncuCyte Live-Cell analysis (D), and the cell colonies were stained with crystal violet staining (E). Images represent one representative experiment from three independent experiments. Data were shown as the mean ± SD. ∗*p* < 0.05; ∗∗*p* < 0.01 versus the control group, Student’s t-test.





**Figure S4. DEHP increased glutamine metabolism in DEHP-exposed cells.** (A-C) The top ten gene sets upregulated by DEHP in (A) biological process (BP), (B) molecular function (MF), and (C) cellular component (CC) were identified in gene ontology (GO) analysis. The size and color of the dots represent the number of genes and the p-adjusted values, respectively. (D) MFF mRNA levels showed no significant change in DEHP-exposed MCF7 cells by RT-qPCR. *n*=3. (E) The protein expressions of mitochondria-related genes in parental and DEHP-exposed MCF7 cells were determined in Western blot with indicated antibodies. (F) DEHP1/MCF7 cells were pretreated with inhibitors against glucose (25 μM 2DG), glutamine (2 mM AOA), and fatty acid (5 μM perhexiline) metabolism for 4 hours. The OCR level was analyzed by Seahorse Metabolic Analyzer following the sequential addition of mitochondrial reagents, oligomycin (1 μM), FCCP (1 μM), and rotenone/ antimycin A (1 μM). Maximal respiration and spare respiratory capacity were calculated. *n*=3. (G) Whole-cell metabolites from parental and DEHP1 MCF7 cells were analyzed with mass spectrometry. (H) MCF7 cells incubated with glutamine free medium for 3 hours were treated with glutaMAX for 2, 4, 6, and 12 hours followed by the measurement of intracellular glutamine levels. *n*=3. Data were shown as the mean ± SD. ns. not significant; ∗*p* < 0.05; ∗∗*p* < 0.01; ∗∗∗*p* < 0.001 versus the control group, Student’s t-test.


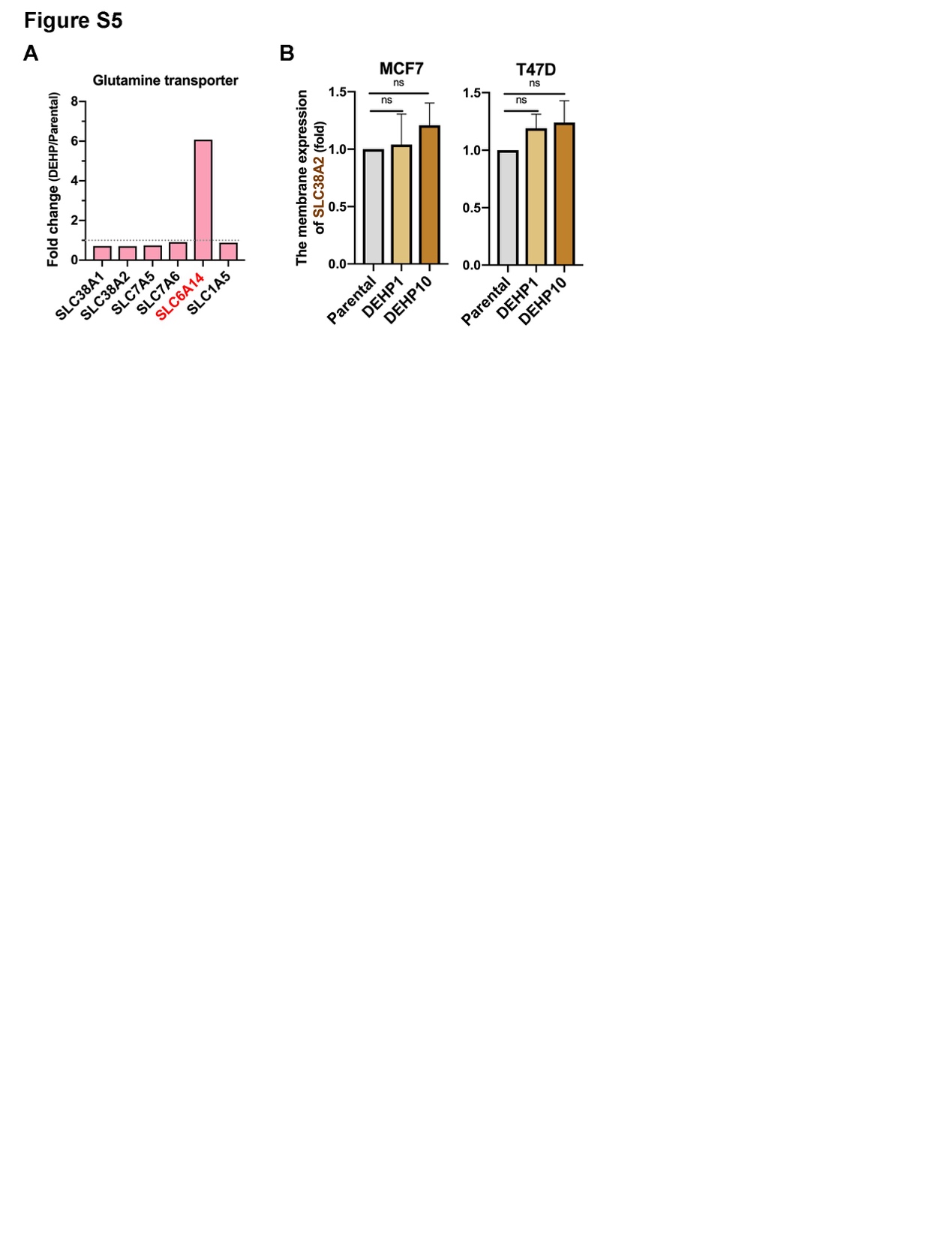


**Figure S5. The impact of DEHP on various glutamine transporters.** (A) The changes in the expression of various glutamine transporters in response to DEHP treatment were analyzed with RNA sequencing data. (B) The expression of SLC38A2 on cell membrane of parental and DEHP-exposed MCF7 and T47D cells was analyzed by flow cytometry. *n*=3. Data were shown as the mean ± SD. ns. not significant versus the control group, one-way ANOVA with Tukey’s multiple comparisons test.





**Figure S6. DEHP upregulates SLC6A14 expression through activation of ERα.**

(A) The positive correlation between the expressions of SLC6A14 and pERα in tumors from total HER2-Tg mice from figure 1D. *n=*10. (B) DEHP-exposed MCF7 and T47D cells were treated with fulvestrant 1, 10, and 100 nM for 48h and subjected to Western blot analysis. (C) The expression of SLC6A14 was reduced in DEHP-exposed MCF7 and T47D cells when ERα was knocked down by infection with shRNA. (D) SLC6A14 mRNA levels in parental and DEHP1 of MCF7 and T47D cells treated with fulvestrant 1, 10, and 100 nM for 2 days were measured by using RT-qPCR. (E) The 3D molecular interaction and 2D binding mode of ligand DEHP, BBP, DBP, and DINP with ERα were analyzed by performing the molecular docking simulation with Discovery Studio software. (F) The protein expressions of SLC6A14 in DEHP, BBP, DBP, and DINP-exposed MCF7 cells were determined in Western blots with the indicated antibodies. Data were shown as the mean ± SD. ∗∗p < 0.01; ∗∗∗p < 0.001 versus the control group, one-way ANOVA with Tukey’s multiple comparisons test.





**Figure S7. Loss of SLC6A14 abrogates DEHP-induced mitochondrial fusion.**

Parental and DEHP-exposed MCF7 cells were transfected with SLC6A14 shRNA. After staining with MitoSpy, the mitochondria morphology was observed using the 3D Cell Explorer-fluo and the average total length of mitochondria was quantified with the Smart Mitochondrial Assay^LIVE^ (Nanolive). Images represent one representative experiment from three independent experiments. Data were shown as the mean ± SD. ns, not significant; ∗*p* < 0.05; ∗∗*p* < 0.01 versus the control group, Student’s t test.





**Figure S8. SLC38A2 is not involved in the DEHP-induced cancer stemness.** (A) Parental and DEHP-exposed MCF7 cells were treated with indicated concentrations of MeAIB for 7 days in spheroid formation assays. (B) The organoid derived from DEHP-exposed HER2-Tg tumors were treated with MeAIB (2.5, 5, and 10 mM) for 7 days in the organoid formation assays. Data were shown as the mean ± SD. ns, not significant; ∗*p* < 0.05 versus the control group, one-way ANOVA with Tukey’s multiple comparisons test.





**Figure S9. Inhibition of SLC6A14 overcomes DEHP-mediated chemoresistance.** (A) ROC analysis of SLC6A14 expression and specificity at five years of relapse-free survival in response to any chemotherapy in ER-positive breast cancer patients. (B) Parental and DEHP-exposed MCF7 cells were treated with indicated concentrations of doxorubicin (Doxo) in combination with/without 0.625mM αMT in a colony formation assay. *n*=3. Data are presented as mean ± SD. Statistical significance was determined by two-way ANOVA. ns, not significant; *p* < 0.05 vs. the control group.


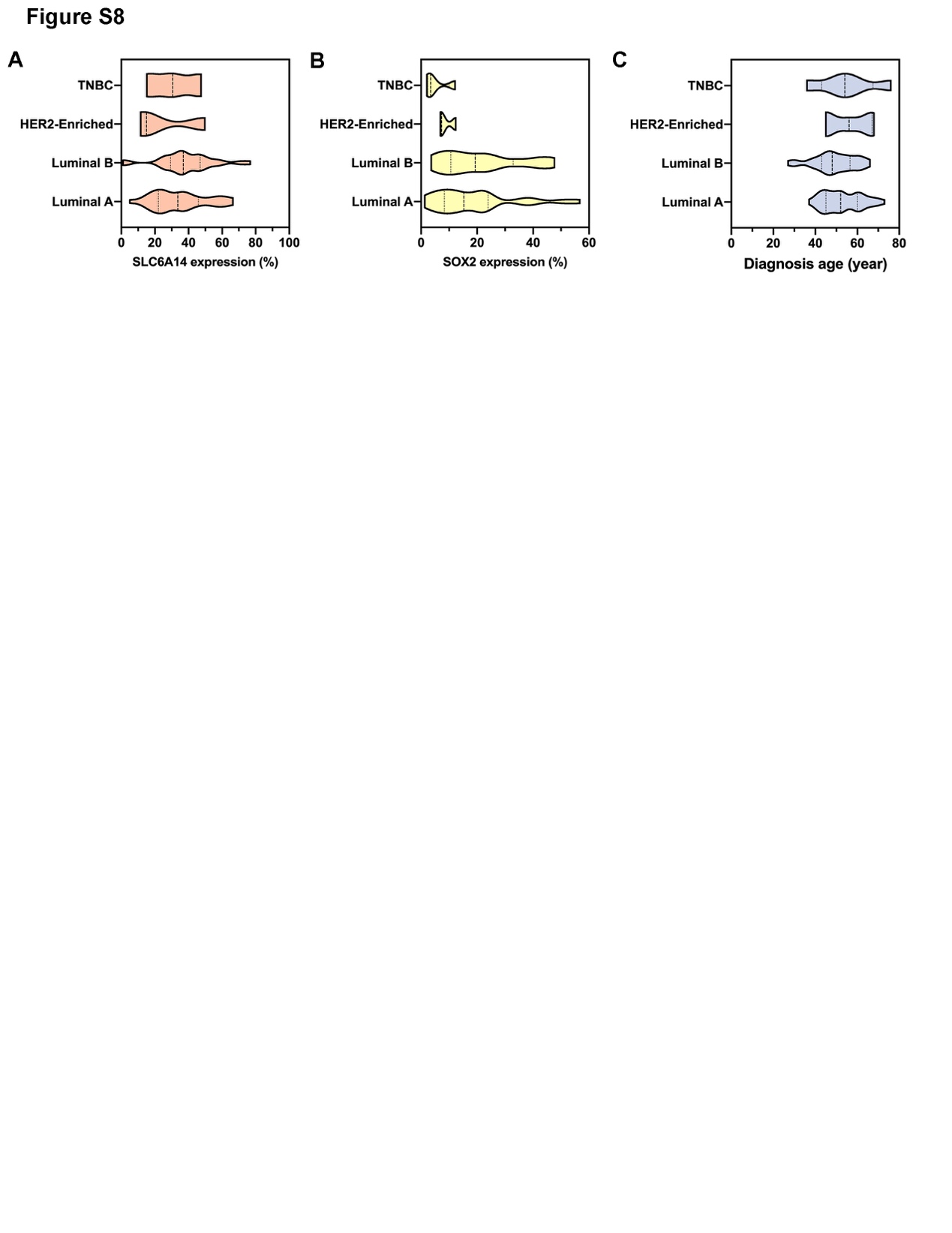


**Figure S10. Expression profiles of SLC6A14 and SOX2 in breast cancer subtypes.** The distributions of (A) SLC6A14 expression, (B) SOX2 expression, and (C) age at diagnosis were analyzed across breast cancer subtypes in this study cohort.

| **Table S1. Associations between SOX2 protein expression and clinical characteristics in breast cancer.** | | | | |
| --- | --- | --- | --- | --- |
| **Variable** | **Cases** | **SOX2 expression** | | ***p*-value** |
|  |  | Low (n=36) | High (n=27) |  |
| **Age (years)** |  |  |  |  |
| ≤45 | 22 (34.9%) | 8 (36.4%) | 14 (63.6%) | 0.015* |
| >45 | 41 (65.1%) | 28 (68.3%) | 13 (31.7%) |  |
| **Molecular subtype** |  |  |  |  |
| Luminal A | 29 (46%) | 16 (55.2%) | 13 (44.8%) | 0.18 |
| Luminal B1 | 18 (28.6%) | 9 (50%) | 9 (50%) |  |
| Luminal B2 | 9 (14.3%) | 4 (44.4%) | 5 (55.6%) |  |
| HER2-enriched | 3 (4.8%) | 3 (100%) | 0 (0%) |  |
| Triple-negative | 4 (6.3%) | 4 (100%) | 0 (0%) |  |
| **Tumor types** |  |  |  |  |
| Ductal carcinoma | 50 (79.4%) | 29 (58%) | 21 (42%) | 0.059 |
| Lobular carcinoma | 2 (3.2%) | 2 (100%) | 0 (0%) |  |
| Mucinous carcinoma | 4 (6.3%) | 0 (0%) | 4 (100%) |  |
| Mixed | 7 (11.1%) | 5 (71.4%) | 2 (28.6%) |  |
| **Tumor grade** |  |  |  |  |
| I | 11 (17.5%) | 5 (45.5%) | 6 (54.5%) | 0.514 |
| II | 40 (63.5%) | 25 (62.5%) | 15 (37.5%) |  |
| III | 12 (19%) | 6 (50%) | 6 (50%) |  |
| **ER status** |  |  |  |  |
| Positive | 56 (88.9%) | 29 (51.8%) | 27 (48.2%) | 0.017* |
| Negative | 7 (11.1%) | 7 (100%) | 0 (0%) |  |
| **PR status** |  |  |  |  |
| Positive | 44 (69.8%) | 21 (47.7%) | 23 (52.3%) | 0.028* |
| Negative | 19 (30.2%) | 15 (78.9%) | 4 (21.1%) |  |
| **HER2 status** |  |  |  |  |
| Positive | 12 (19%) | 7 (58.3%) | 5 (41.7%) | 0.926 |
| Negative | 51 (81%) | 29 (56.9%) | 22 (43.1%) |  |
| **Ki67 status** |  |  |  |  |
| High | 32 (50.8%) | 19 (59.4%) | 13 (40.6%) | 0.716 |
| Low | 31 (49.2%) | 17 (54.8%) | 14 (45.2%) |  |
| **E-cadherin status** |  |  |  |  |
| Positive | 8 (12.7%) | 4 (50%) | 4 (50%) | 0.715 |
| Negative | 55 (87.3%) | 32 (58.2%) | 23 (41.8%) |  |
| **Recurrence** |  |  |  |  |
| Yes | 8 (12.7%) | 7 (87.5%) | 1 (12.5%) | 0.123 |
| No | 55 (87.3%) | 29 (52.7%) | 26 (47.3%) |  |
| Pearson chi-square analysis was used for discrete variables. *: p-value <0.05.  ^#^ Molecular subtypes: Luminal A (HR^+^, HER2^-^, Ki67 low); Luminal B1 (HR^+^, HER2^-^, Ki67 high); Luminal B2 (HR^+^, HER2^+^, any Ki67); HER2-enriched (HR^-^, HER2^+^, any Ki67); Triple-negative (HR^-^, HER^-^, any Ki67). | | | | |

| **Table S2. Odd ratio analysis for SOX2 protein expression in early onset of breast cancer.** | | | | |
| --- | --- | --- | --- | --- |
| Variable | **SOX2 expression** | | **OR (95%CI)** | ***p*-value** |
|  | High (n=27) | Low (n=36) |  |  |
| Age (years) |  |  |  |  |
| ≤45 | 14 (63.6%) | 8 (36.4%) | 3.760 (1.268-11.206) | 0.015 |
| >45 | 13 (31.7%) | 28 (68.3%) |  |  |
| Data were calculated using unconditional logistic regression.  ER, estrogen receptor; PR, progesterone receptor; OR, odds ratio; CI, confidence interval, N/A, not available. | | | | |

| **Table S3. Associations between SLC6A14 protein expression and clinical characteristics in breast cancer.** | | | | |
| --- | --- | --- | --- | --- |
| **Variable** | **Cases** | **SLC6A14 expression** | | ***p*-value** |
|  | (n=67) | Low (n=26) | High (n=41) |  |
| **Age (years)** |  |  |  |  |
| ≤45 | 21 (31.3%) | 4 (19%) | 17 (81%) | 0.025^*^ |
| >45 | 46 (68.7%) | 22 (47.8%) | 24 (52.2%) |  |
| **Molecular subtype** |  |  |  |  |
| Luminal A | 31 (46.2%) | 15 (48.4%) | 16 (51.6%) | 0.312 |
| Luminal B1 | 19 (28.4%) | 4 (21%) | 15 (78.9%) |  |
| Luminal B2 | 11 (16.4%) | 4 (36.4%) | 7 (63.6%) |  |
| HER2-enriched | 3 (4.5%) | 2 (66.7%) | 1 (33.3%) |  |
| Triple-negative | 3 (4.5%) | 1 (33.3%) | 2 (66.7%) |  |
| **Tumor types** |  |  |  |  |
| Ductal carcinoma | 53 (79.1%) | 21 (39.6%) | 32 (60.4%) | 0.38 |
| Lobular carcinoma | 2 (3%) | 1 (50%) | 1 (50%) |  |
| Mucinous carcinoma | 4 (6%) | 0 (0%) | 4 (100%) |  |
| Mixed | 8 (11.9%) | 4 (50%) | 4 (50%) |  |
| **Tumor grade** |  |  |  |  |
| I | 12 (17.9%) | 3 (25%) | 9 (75%) | 0.388 |
| II | 45 (67.2%) | 20 (44.4%) | 25 (55.6%) |  |
| III | 10 (14.9%) | 3 (30%) | 7 (70%) |  |
| **ER status** |  |  |  |  |
| Positive | 61 (91%) | 23 (37.7%) | 38 (62.3%) | 0.67 |
| Negative | 6 (9%) | 3 (50%) | 3 (50%) |  |
| **PR status** |  |  |  |  |
| Positive | 49 (73.1%) | 19 (38.8%) | 30 (61.2%) | 0.993 |
| Negative | 18 (26.9%) | 7 (38.9%) | 11 (61.1%) |  |
| **HER2 status** |  |  |  |  |
| Positive | 14 (20.9%) | 6 (42.9%) | 8 (57.1%) | 0.727 |
| Negative | 53 (79.1%) | 20 (37.7%) | 33 (62.3%) |  |
| **Ki67 status** |  |  |  |  |
| High | 33 (49.3%) | 9 (27.3%) | 24 (72.7%) | 0.056 |
| Low | 34 (50.7%) | 17 (50%) | 17 (50%) |  |
| **E-cadherin status** |  |  |  |  |
| Positive | 10 (14.9%) | 3 (30%) | 7 (70%) | 0.729 |
| Negative | 57 (85.1%) | 23 (40.4%) | 34 (59.6%) |  |
| **Recurrence** |  |  |  |  |
| Yes | 7 (10.4%) | 1 (14.3%) | 6 (85.7%) | 0.234 |
| No | 60 (89.6%) | 25 (41.7%) | 35 (58.3%) |  |
| Pearson chi-square analysis was used for discrete variables. *: p-value <0.05.  ^#^ Molecular subtypes: Luminal A (HR^+^, HER2^-^, Ki67 low); Luminal B1 (HR^+^, HER2^-^, Ki67 high); Luminal B2 (HR^+^, HER2^+^, any Ki67); HER2-enriched (HR^-^, HER2^+^, any Ki67); Triple-negative (HR^-^, HER^-^, any Ki67). | | | | |

| **Table S4. Odd ratio analysis for SLC6A14 protein expression in early onset of breast cancer.** | | | | |
| --- | --- | --- | --- | --- |
| Variable | **SLC6A14 expression** | | **OR (95%CI)** | ***p*-value** |
|  | High (n=41) | Low (n=26) |  |  |
| Age (years) |  |  |  |  |
| ≤45 | 17 (81%) | 4 (19%) | 3.896 (1.135-13.372) | 0.025 |
| >45 | 24 (52.2%) | 22 (47.8%) |  |  |
| Data were calculated using unconditional logistic regression. OR, odds ratio; CI, confidence interval. | | | | |

| **Table S5. Odd ratio analysis for SLC6A14 expression in SOX2 cancer stemness marker expression in breast cancer.** | | | | | |
| --- | --- | --- | --- | --- | --- |
| SOX2 expression | **Cases (n=59)** | **SLC6A14 expression** | | **OR**  **(95%CI)** | ***p*-value** |
|  |  | High (n=38) | Low (n=21) |  |  |
| High | 27 (45.8%) | 22 (57.9%) | 5 (23.8%) | 4.400  (1.335-14.506) | 0.012 |
| Low | 32 (54.2%) | 16 (42.1%) | 16 (76.2%) |  |  |
| Pearson chi-square analysis was used for discrete variables.  Data were calculated using unconditional logistic regression. OR, odds ratio; CI, confidence interval. | | | | | |
